# Quantifying the Hydration‐Dependent Dynamics of Cu Migration and Activity in Zeolite Omega for the Partial Oxidation of Methane

**DOI:** 10.1002/anie.202407395

**Published:** 2024-11-02

**Authors:** Johannes Wieser, Dariusz Wardecki, Jörg W. A. Fischer, Mark A. Newton, Catherine Dejoie, Amy J. Knorpp, Thomas C. Hansen, Gunnar Jeschke, Przemyslaw Rzepka, Jeroen A. van Bokhoven

**Affiliations:** ^1^ Department of Chemistry and Applied Biosciences, Institute for Chemical and Bioengineering ETH Zurich 8093 Zürich Switzerland; ^2^ Institute of Experimental Physics, Faculty of Physics University of Warsaw 02-093 Warsaw Poland; ^3^ Department of Chemistry and Applied Biosciences, Institute of Molecular Physical Science ETH Zurich 8093 Zürich Switzerland; ^4^ Department of Structure and Dynamics in Catalysis J. Heyrovsky Institute of Physical Chemistry Dolejškova 2155/3 182 23 Prague 8 Czech Republic; ^5^ ID22 European Synchrotron Radiation Facility 38043 Grenoble France; ^6^ Institut Laue-Langevin 71 Avenue des Martyrs 38000 Grenoble France; ^7^ Center for Energy and Environmental Science Paul Scherrer Institute (PSI) 5232 Villigen Switzerland

**Keywords:** Anomalous X-ray Diffraction, Copper Active Sites, Zeolite, EPR Spectroscopy, Methane, Neutron Diffraction

## Abstract

Copper‐exchanged zeolite omega (Cu‐omega) is a potent material for the selective conversion of methane‐to‐methanol (MtM) via the oxygen looping approach. However, its performance exhibits substantial variation depending on the operational conditions. Under an isothermal temperature regime, Cu‐omega demonstrates subdued activity below 230 °C, but experiences a remarkable increase in activity at 290 °C. Applying a high‐temperature activation protocol at 450 °C causes a rapid deactivation of the material. This behavioral divergence is investigated by combining reactivity studies, neutron diffraction and in situ high‐resolution anomalous X‐ray powder diffraction (HR‐AXRPD), as well as electron paramagnetic resonance spectroscopy, to reveal that the migration of Cu throughout the framework is the primary cause of these behaviors, which in turn is predominantly governed by the degree of hydration of the system. This work suggests that control over the Cu migration throughout the zeolite framework may be harnessed to significantly increase the activity of Cu‐omega by generating more active sites for the MtM conversion. These results underscore the power of in situ HR‐AXRPD for unraveling the behavior of materials under reaction conditions and suggest that a re‐evaluation of Cu‐zeolites priorly deemed inactive for the MtM conversion across a broader range of conditions and looping protocols may be warranted.

## Graphical abstract

Cu‐exchanged zeolite omega's (Cu‐omega) activity in partial methane oxidation via oxygen looping significantly varies with the conditions applied. Using primarily in situ anomalous x‐ray powder diffraction (AXRPD) the source of this divergent behavior is revealed to be due to a migration of Cu throughout the framework, which in turn is predominantly controlled by the system's degree of hydration.

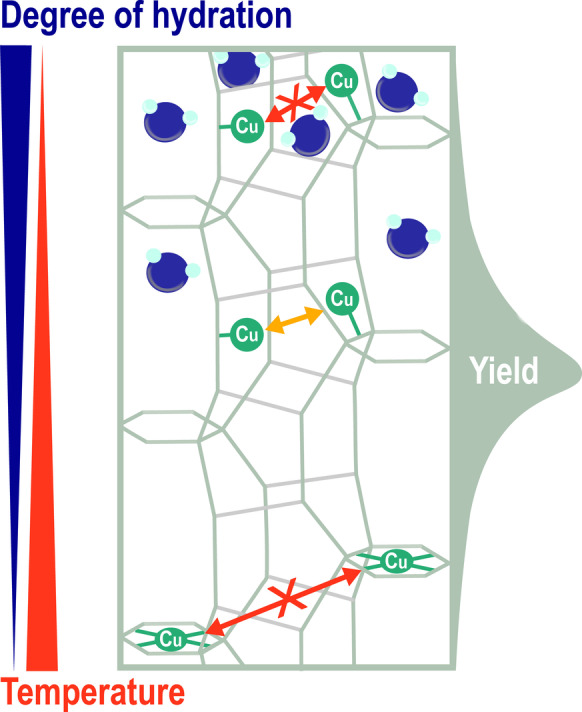

## Introduction

Methane is a common feedstock molecule for the synthesis of a wide array of chemicals, as well as being used directly as a fuel itself. A significant amount of methane is, however, flared to yield carbon dioxide and water every year. This wasteful and polluting process is often performed at remote oil exploitation sites to avoid the emission of methane into the atmosphere. The rationale for flaring is twofold: the global warming impact of methane significantly surpasses that of carbon dioxide;^[1]^ and the economies‐of‐scale do not permit the use of those extant processes that can convert methane into a more readily transportable product, such as methanol, at such locations.[^2^, ^3^] The latter reason for flaring is due to the nature of the conventional process, which involves two highly energy intensive steps; the conversion of methane into syngas,^[4]^ followed by a conversion of syngas into methanol.[^2^, ^3^, ^5^] The high energy and capital investment costs make the indirect, two‐step, conversion process economically unfeasible at small scales and remote locations.[^2^, ^6^, ^7^]

This issue necessitates the development of a scale‐flexible and direct approach to the conversion of methane‐to‐methanol (MtM). However, all direct conversion pathways are subject to intrinsically unfavorable selectivity‐conversion limits caused by the stability of methane and the comparative instability of methanol under the conditions necessary to activate methane.[^8^, ^9^, ^10^, ^11^] To avoid this limitation, methanol needs to be protected from subsequent oxidation.^[11]^ One potential solution is the oxygen looping process, a physio‐chemical protection strategy.[^12^, ^13^, ^14^] This process entails three consecutive steps:[^2^, ^5^] activation of the material under an oxidant; reaction with methane to form a surface bound methoxy intermediate; desorption of the product methanol using steam.

A class of material investigated extensively for the direct MtM conversion are Cu‐exchanged zeolites.[^12^, ^15^] Zeolites are crystalline microporous solids constructed of tetrahedrally coordinated silicon and aluminum atoms, bridged by oxygen atoms.^[16]^ These so‐called primary building units can be arranged in a multitude of ways to produce zeolites with markedly different characteristics, such as pore size, channel dimensionality and Si/Al ratios.^[17]^ The negative charge created by replacing a framework silicon with a framework aluminum needs to be counterbalanced by an extra‐framework cation. If the cation is a proton, it creates a Brønsted acid site. The introduction of alternative cations that can exhibit various oxidation states, such as Cu, may cause the material to be active for redox reactions.^[18]^ This, in tandem with the excellent thermal stability offered by zeolites, makes Cu‐exchanged zeolites popular candidates for redox reactions.^[18]^ One notable example is the use of Cu‐zeolites for the removal of NOx gases by the selective catalytic reduction with ammonia (NH_3_‐SCR).[^19^, ^20^, ^21^, ^22^, ^23^]

This widespread application of Cu‐zeolites, along with their potential for alternative processes such as the direct MtM conversion reaction,^[12]^ has resulted in significant amounts of research being directed toward understanding these materials. The location,[^22^, ^24^, ^25^, ^26^, ^27^] nuclearity[^28^, ^29^] and activity^[30]^ of the Cu active sites for different potential reactions, as well as the potential for migration of the Cu itself throughout the framework,[^19^, ^20^, ^21^, ^29^, ^31^, ^32^, ^33^, ^34^] are just some of the characteristics of these materials that have been investigated for over 30 years. These characteristics are in turn dependent on factors such as the aluminum content and distribution throughout the framework, the Cu/Al ratio, as well as the topology of the framework itself.^[28]^ This cascade of interdependent characteristics in Cu‐zeolites has necessitated the use of a broad range of methods to investigate the mechanisms that govern these characteristics, as well as assess their impact on a given redox‐reaction.

Since zeolite‐type catalysts are powders, the traditional tool for determining their crystal structures is X‐ray powder diffraction (XRPD). Over the past 20 years, notable advancements have been made in using XRPD to determine the location and configuration of extra‐framework Cu inside zeolitic frameworks.[^22^, ^24^, ^26^, ^27^] Cu‐exchanged zeolites have been shown to host Cu in a variety of configurations and nuclearities to yield sites that exhibit different efficacies and mechanisms for the conversion of methane.[^28^, ^30^, ^35^, ^36^, ^37^] In contrast to spectroscopic and computational methods, XRPD enables the direct identification of the positions of Cu sites.[^24^, ^33^] Nevertheless, the refinements are often challenging due to the sparse amounts of Cu closely associated with other elements.[^25^, ^38^] To tackle this problem, we have successfully utilized anomalous X‐ray powder diffraction (AXRPD), which exploits the changes in X‐ray atomic factors near the atomic absorption edges, to highlight the signals at framework^[39]^ and non‐framework^[40]^ positions. In comparison to XRPD, AXRPD therefore allows for a definitive distinction between the signals of different elements. This makes AXPRD at the Cu K‐edge a powerful tool for determining the location and configuration of Cu sites within a zeolite lattice. However, the nature of the active site may change significantly under reaction conditions. This makes in situ and operando studies of these materials essential. A material, which due to its exceptional activity toward the NH_3_‐SCR reaction has been the focus of multiple XRPD studies, often in conjunction with spectroscopic methods, is Cu‐exchanged SSZ‐13 (CHA).[^25^, ^31^, ^32^, ^33^, ^41^] Andersen et al.^[33]^ and Beale et al.^[41]^ unveiled a migration of the Cu between two distinct crystallographic positions in the structure as a function of temperature, and correspondingly, degree of hydration. This research has further demonstrated that Cu at these two positions exhibits different degrees of activity toward the NH_3_‐SCR reaction.^[41]^ The mobility of Cu in SSZ‐13 is not solely reduced to a movement between 8 MR and 6 MR positions. Dinh et al. investigated Cu‐SSZ‐13 for the MtM conversion and determined that Cu may migrate throughout the whole framework.^[42]^

**Scheme 1:**
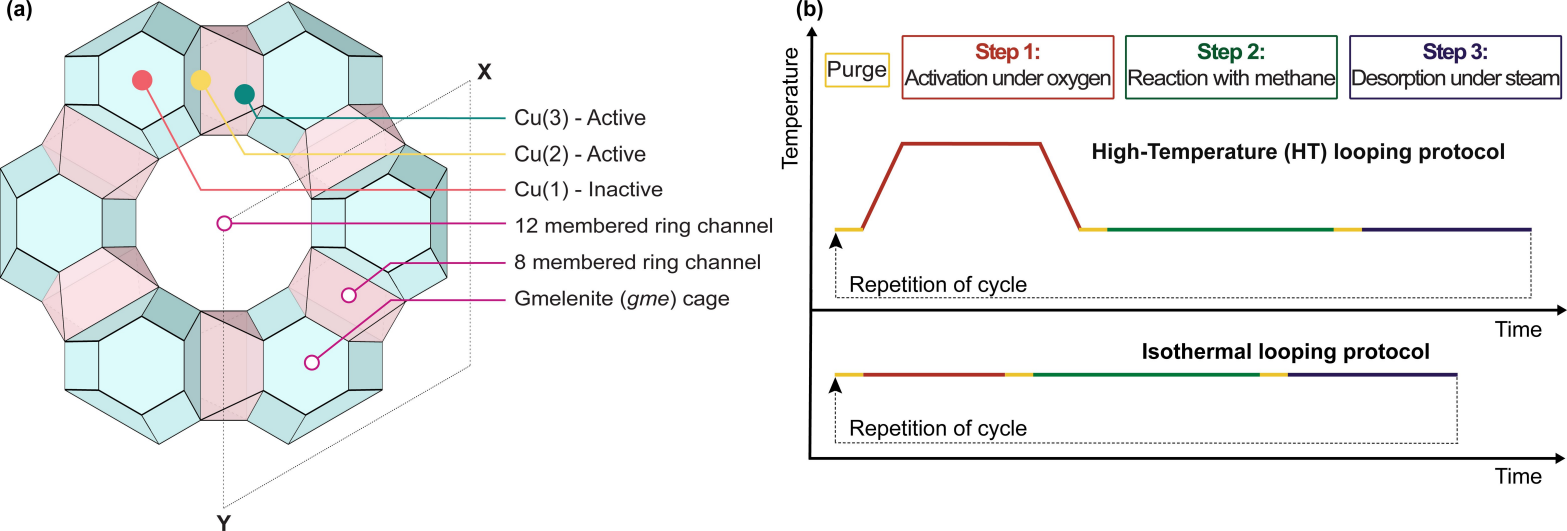
**(a)** The structure of zeolite omega, depicted along [001], showing the 12 and 8 MR channels, as well as the characteristic *gme* cages. As well depicted are the observed Cu‐positions, which are solely accessible via the 8 MR channels **(b)** Schematic representation of the high‐temperature (HT) activation and isothermal activation protocol.

Among the numerous Cu‐zeolites examined for the MtM conversion, Cu‐exchanged zeolite omega (MAZ framework topology) has recently emerged as one of the most active and selective materials within the oxygen looping paradigm.[^5^, ^40^, ^43^, ^44^, ^45^, ^46^] The framework structure of zeolite omega is hexagonal, belonging to the P*63*/*mmc* space group. The structure consists of gmelenite (*gme*) cavities stacked in a zigzag manner and interconnected via 8 membered rings (MR). These are arranged around the 12 MR channels. The 8 MR apertures of the *gme* cages form the winding 8 MR channels, organized by a *c*‐glide plane. In zeolite omega, the 8 MR and 12 MR systems are completely isolated from each other. This is depicted in Scheme 1a.^[47]^

Prior research on Cu‐omega has shown that the reaction proceeds via a two‐electron redox mechanism.[^30^, ^40^, ^46^] The active site has been determined to be a pair of proximal Cu−OH monomers situated in adjacent 8 MR entrances to the *gme* cages (Cu(2) and Cu(3)), with isolated inactive Cu positioned in the plane of the 6 MR aperture between the neighboring cages (Cu(1)).^[40]^ Each active 8 MR Cu monomer charge balances one aluminum site of the zeolite framework, while each inactive 6 MR Cu balances two framework aluminum sites.^[40]^ The determination of the different Cu positions was achieved via ex situ AXRPD after the sample was activated under oxygen at 450 °C. However, the refinement did show that the interatomic distances between two proximal Cu atoms are not distant enough to exclude an oxygen bridged dimeric active site. Therefore, ex situ diffuse reflectance UV/Vis spectroscopy was used to rule out the presence of such dimers, and to show that the active sites are of purely monomeric nature under the conditions examined.^[40]^

In this work we have investigated the efficacy of Cu‐omega for both the high‐temperature (HT) activation[^43^, ^44^, ^48^] and isothermal oxygen looping approaches.[^7^, ^19^, ^20^, ^21^, ^26^] The HT approach entails an aerobic activation at a temperature of 450 °C, followed by the reaction and steaming steps at a temperature of 200 °C.^[48]^ The isothermal approach is generally performed in a temperature range of 200–300 °C^[5]^ and avoids time‐ and energy‐intensive heating and cooling ramps.[^5^, ^48^, ^49^] This is advantageous for a prospective industrial‐scale application, which must meet specific criteria in order to be cost‐effective, which includes a minimum productivity of 3000 μmol g_zeolite_^−1^ h^−1^.^[2]^ Prior work has focused on understanding the kinetics of each step of the looping cycle, and has demonstrated that an appropriate reduction of the timeframes of a single cycle is where the most significant benefit to productivity may be achieved.^[4]^ Therefore, the isothermal looping protocol is preferable to the HT‐activation protocol.^[5]^ A schematic of both protocols is depicted in Scheme 1b.

Cu‐omega has been shown to be highly active in both the HT‐activation and isothermal looping protocols.[^5^, ^43^, ^44^, ^45^, ^48^] However, prior work has shown that performing a second cycle with Cu‐omega via the HT‐activation protocol results in the yield decreasing to ~1/2 of the first cycle, though with no apparent loss of framework stability.^[43]^ Conversely, using the isothermal looping approach at temperatures between 250–300 °C leads to an increase in yield over consecutive cycles.^[5]^ Using a temperature below 250 °C results in comparatively low yields.^[5]^

To enhance the methanol productivity of transition metal loaded zeolites, the number of active sites and the rate of reaction of each individual step must be maximized. It is crucial to understand the characteristics and interplay of the active site and zeolitic structure. This may vary significantly based on factors like structural topology and the Si/Al ratio.^[28]^ Understanding the behavioral variation of the Cu cations with respect to the zeolite structure, as well as varying reaction conditions and looping regimes, is essential for enhancing Cu‐omega's potential for the partial oxidation of methane. In situ high‐resolution AXRPD at the Cu K‐edge provides a clear identification of different Cu positions, as well as a monitoring of any displacement of the Cu within the zeolite framework. When combined with Cu K‐edge X‐ray Absorption Near Edge Structure (XANES), in situ Electron Paramagnetic Resonance (EPR) spectroscopy and reactivity studies, the profound impact of different reaction conditions on the Cu location within the zeolite framework, and the corresponding performance of the material for the MtM conversion, becomes evident. This highlights the importance of a precise mapping of the structure–activity relationship in materials as complex as Cu‐zeolites. The extreme condition‐dependent variation in material activity suggests that a re‐examination of Cu‐zeolites deemed inactive for methane partial oxidation may uncover previously untapped potential.

## Results & Discussion

**Figure 1:**
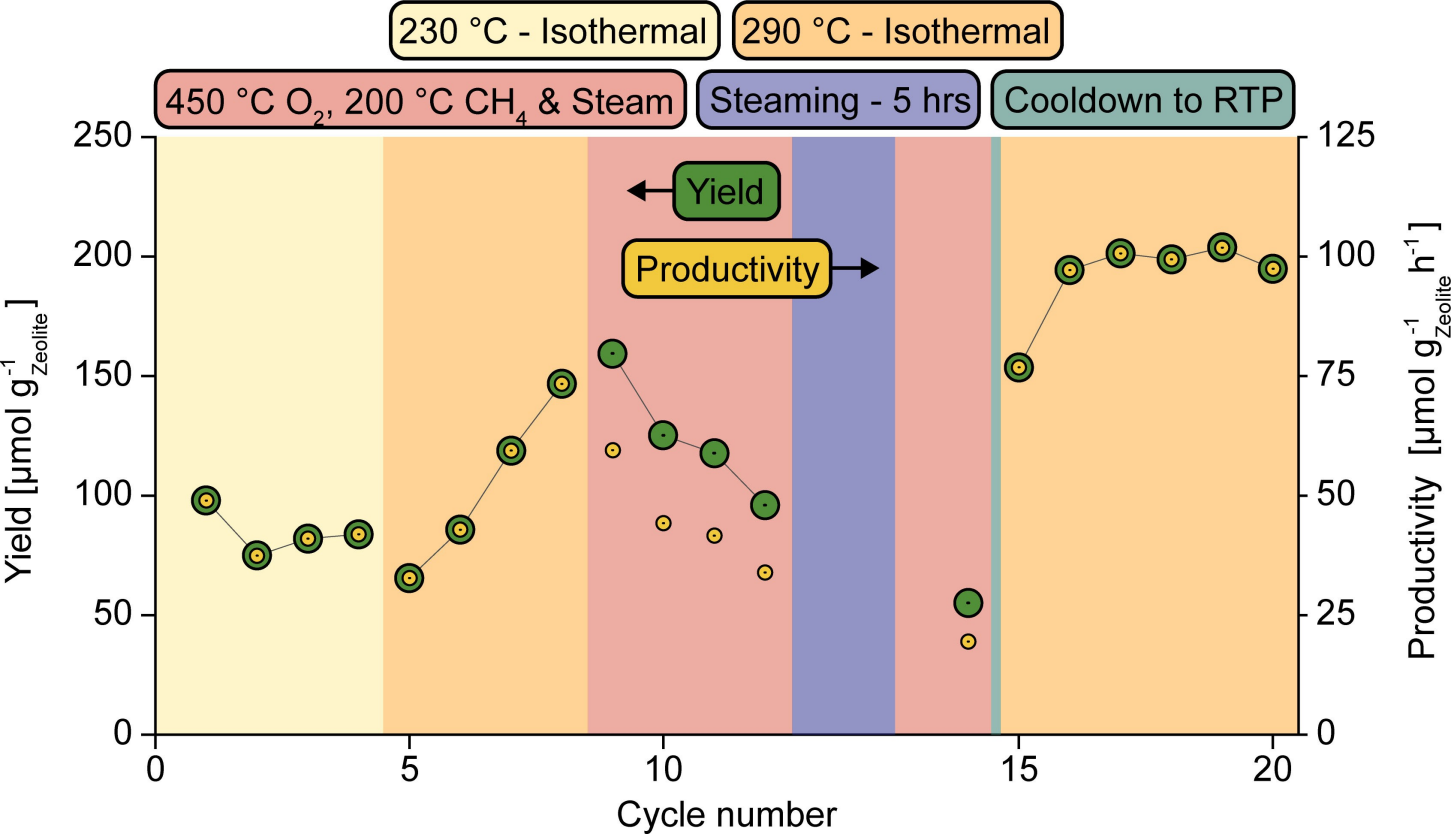
Change in yield, on a methanol equivalent basis, as well as the productivity, as a function of temperature and looping procedure. The legend and color Scheme describe what conditions the material is exposed to at the observed values of yield and productivity. The depicted results for productivity are significantly lower than in prior work.^[5]^ This discrepancy arises as the productivity was calculated using the total exposure time under each reactant, rather than the actual much shorter necessary timeframes as determined by operando X‐ray absorption spectroscopy.^[5]^

The efficacy of Cu‐omega in respect to the MtM conversion varies significantly depending on the chosen conditions. Prior work has also shown that performing consecutive cycles may have a significant effect on the material's activity toward MtM conversion.^[5]^ Therefore, the effect of consecutive cycling was investigated by performing multiple cycles on the same material at various conditions. The results are depicted in Figure 1.

Applying the isothermal protocol in the region of 200–250 °C results in low activity for Cu‐omega toward the MtM conversion.^[5]^ To examine the effect of performing multiple cycles on the activity of Cu‐omega in this temperature region, four consecutive cycles at 230 °C were performed. Very similar yields of ~80 μmol g_zeolite_^−1^ for each cycle are observed, with the first cycle yielding a slightly higher amount than the following ones. Increasing the temperature to 290 °C causes a significant increase in the observed yield and corresponding productivity with each consecutive cycle. Following this, four cycles using the HT‐activation approach were performed. The first cycle exhibited a higher yield than prior cycles, followed by a decrease in yield over the following cycles. A similar trend has been observed for Cu‐omega in previous studies.^[43]^ However, the values for productivity decrease when switching from an isothermal to a HT‐activation protocol, even in the case of the first HT cycle, where an enhanced yield to previous cycles was observed. . This is due to the additional time needed for the applied heating and cooling steps under an oxygen atmosphere, as may be seen in Scheme 1b.

To see if prolonged steaming has an influence on the yield of the following loops, the sample was exposed to wet helium for five hours at 200 °C. Post‐steaming, two cycles using the HT‐protocol were performed. The second cycle of the HT‐protocol exhibited the lowest yield of any cycle observed in the whole measurement. This shows that prolonged steaming is not beneficial in respect to the yield when using the HT‐activation protocol in the long run. The material was then cooled down to room temperature under a flow of dry helium. The sole reason for cooling to room temperature was to reset the reactor macros.

Following the HT‐activation cycles and cooling to room temperature, the sample was once again heated to 290 °C under oxygen, and six cycles following the isothermal looping protocol were performed. The yield achieved in the first cycle post HT‐activation protocol is similar in yield to the last cycle pre HT‐activation protocol, both at 290 °C, at 154 μmol g_zeolite_^−1^ and 147 μmol g_zeolite_^−1^ respectively. In both cases the Cu‐usage is ~0.4. The subsequent cycles all exhibit even greater yields of around ~200 μmol g_zeolite_^−1^ (~100 μmol g_zeolite_^−1^ h^−1^), Cu‐usage ~0.6, Figure S9). By comparison, all prior cycles at 290 °C, as well as the cycles performed using the HT‐activation approach, had shown a significant variation in yield. Furthermore, the value of ~200 μmol g_zeolite_^‐1^ is the highest yield achieved in this cycling sequence. This shows that the deactivation of Cu‐omega can be reversed when appropriate conditions are applied.

**Figure 2:**
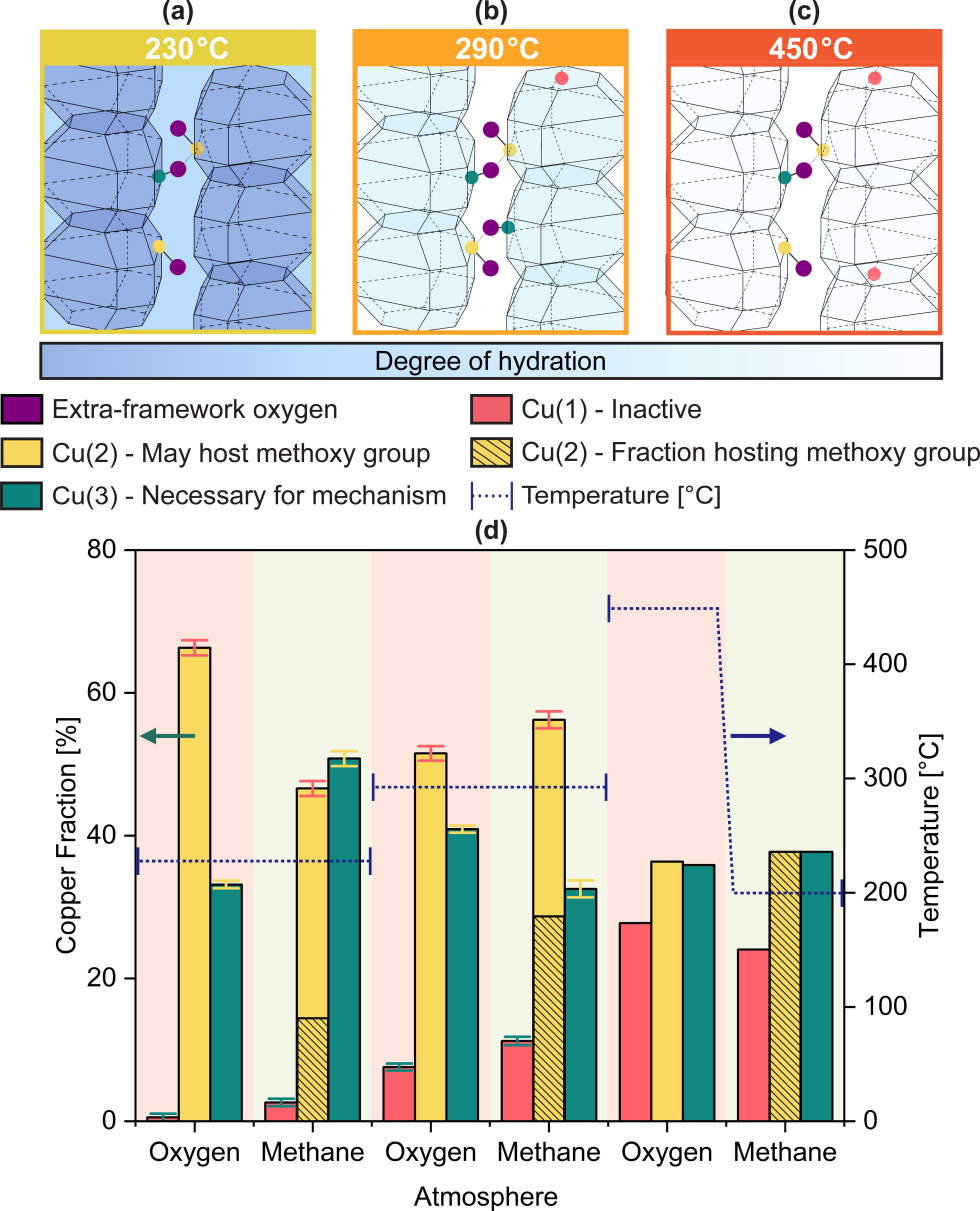
Arrangement of *gme* cavities around the 8 MR *c*‐glide channels and observed positions of Cu cations at different temperatures and hydration levels: **(a)** 230 °C, and at the highest hydration level, site Cu(1) is empty; **(b)** at 290 °C, significant amounts of water remain in the *gme* cages **(c)** at 450 °C and full dehydration. **(d)** Cu‐occupancies of the three observed Cu positions as a percentage of the total Cu population per unit cell under different conditions. The temperature the material is exposed to is indicated by the dotted blue line. The fraction of Cu(2) hosting a methoxy group at the respective conditions is represented with shaded lines. Data for 230 °C and 290 °C using the isothermal protocol were collected in situ at ID22 of the ESRF, while data for the 450 °C HT‐protocol were collected ex situ at the MS beamline of the SLS by Knorpp et al.^[40]^ The total Cu present in the system in the case of data collected at ID22 remains in the three‐sigma level deviation from the mean (Figure S10). The uncertainties were evaluated during the Rietveld analysis of the data using the Singular Value Decomposition method implemented in the Topas Academic software.^[50]^

To determine the source of the observed variation in activity that Cu‐omega exhibits at various conditions, the material was further investigated by monitoring the Cu location via in situ HR‐AXRPD coupled with in situ XANES spectroscopy in fluorescence mode. Difference Fourier maps (DFM) calculated using on‐ and off‐resonance diffraction data permit the resolution of the residual electron densities generated by different elements. Cu can be unequivocally assigned to three distinct density peaks. Subsequent Rietveld analysis shows that the Cu occupation of these sites is temperature dependent. The possible locations, as well as the occupancies of the various Cu species under different conditions are depicted in Figure 2.

Figure 2a–c depicts the observed Cu locations inside the framework of zeolite omega. Three preferred Cu positions are observed; two positions within the 8 MR *c*‐glide channels, labelled Cu(2) and Cu(3), and a third (Cu(1)) centered in the 6‐rings of the *gme* cages. Rietveld analysis of the XRPD data further shows that the methoxy group intermediate is exclusively hosted on the Cu(2) position.

Upon activation under oxygen, both Cu(2) and Cu(3) monomers exhibit −OH groups. Both groups are modeled by the extra‐framework position O(10). Cu(2) and Cu(3) occupy adjacent 8 MR's and constitute the active site for the MtM conversion. As shown in Figure 2d, at 230 °C under an oxygen atmosphere, the 6 MR of the *gme* cages remains unoccupied. A schematic representation of the Cu locations at 230 °C is depicted in Figure 2a. A significant disparity between the amounts of Cu(2) and Cu(3), both located in the 8 MR apertures of the *gme* cages, may also be observed. This disparity between Cu(2) and Cu(3), at ~66 % and 33 % of the total Cu present shows that not all the monomers exist as a pair at this temperature.

Switching to a methane atmosphere causes an increase in the population of inactive Cu(1) (Figure 2d). However, the increase is not very pronounced (~3 % of the total Cu). At 230 °C large amounts of water remain in the system and may occupy the *gme* cages. Linear combination analysis of the simultaneously collected Cu K‐edge XANES (Figure S6) shows that a significant fraction of the Cu remains present in hydrated form. Most of the Cu migration occurs between the two 8 MR positions of the *gme* cages. Cu(3) increases from ~33 % to 51 %, while Cu(2) decreases from ~66 % to 47 %. The resulting populations of the two 8 MR Cu positions become almost equivalent. The DFMs and refinement show that Cu(3) under these conditions is split into two symmetry‐equivalent positions, and that a slight shift of the Cu toward the center of the channel has taken place. The refinement further shows that only a third of all Cu(2), corresponding to 14 % of the total Cu, hosts a methoxy group. As two Cu's are needed per methoxy intermediate, ~68 % of all Cu, while being present in the 8 MR, remains inactive at this temperature. An oxygen will remain coordinated to all Cu(3), as well as to Cu(2) that has not reacted with methane. In the case of unreacted Cu(2) and Cu(3), this oxygen belongs to the hydroxy groups. For a Cu(3) adjacent to a Cu(2) harboring a methoxy group, we ascribe this to a water molecule, as per the suggested mechanism.[^5^, ^40^] After methane, the material was exposed to steam to desorb the product methanol (Scheme 1b), although this was not monitored by diffraction tools due to the presence of significant amounts of water in the system.

A further cycle was performed after heating the sample to 290 °C under an oxygen atmosphere. An increase in the population of inactive Cu(1) is observed (Figure 2d). When comparing the occupancy of Cu(1) under oxygen atmospheres at 230 °C and 290 °C, an increase from 0 % to ~8 % of the total Cu is observed. Concurrently, the occupation of Cu(2) increases to ~52 %, whilst that for Cu(3) drops to 41 %. Switching to a methane atmosphere causes a further increase in Cu(1) to ~11 %, and Cu(2) to ~56 % of the total Cu present in the system. The occupancy of Cu(3) decreases to ~33 %. As such, the disparity between Cu(2) and Cu(3) increases when switching from oxygen to methane at 290 °C, whereas at 230 °C the opposite was observed. The increase in inactive Cu(1), in comparison to results at 230 °C under a methane atmosphere proceeds at the expense of potentially active 8 MR Cu. At a temperature of 290 °C, almost all Cu will be present in its dehydrated form, as shown by the simultaneously collected Cu K‐edge XANES (Figure S6) and prior investigations on Cu‐omega.^[5]^ Under these conditions 51 % of all Cu(2), equating to ~29 % of the total Cu, hosts a methoxy group. The fraction of Cu(2) hosting a methoxy group and Cu(3), at ~29 % and ~33 %, are very similar, suggesting that most of Cu(3) will be part of a paired monomer active site. All Cu(3) is bound to an oxygen, which according to the mechanism belongs to a water molecule formed during the reaction.[^5^, ^40^] The remaining ~28 % Cu(2) is present in the 8 MR as lone monomers, which once more remain inactive toward methane activation.

An increase in temperature to 450 °C under oxygen causes a significant increase in Cu(1) to 28 % at the expense of the 8 MR active sites. A significant decrease in Cu(2) from ~56 % to ~36 %, along with a slight increase in Cu(3) from ~33 % to 36 %, is observed, when compared to the values previously obtained under methane at 290 °C. The levels of Cu(2) and Cu(3) occupancy attain an essential parity. Almost all the Cu present in the 8 MR openings therefore may potentially comprise an active paired monomer site. The HT‐activation is followed by a cooling to 200 °C, and an exposure to a methane atmosphere. Interestingly, a small decrease in Cu(1) (~28 % to ~24 %), and a slight increase in both Cu(2) and Cu(3) is observed, with the levels of the latter two remaining approximately equal. All Cu(2) is hosting a methoxy group, thereby suggesting that all Cu remaining in the 8 MR will be in a paired monomer configuration. Once again, an oxygen belonging to a water molecule is bound to all Cu(3).

**Figure 3:**
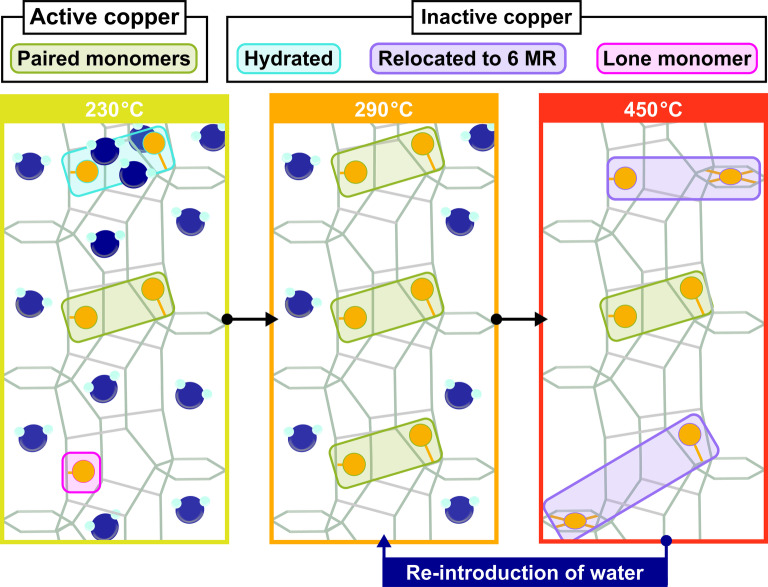
Schematic illustration of the various observed Cu configurations, and their corresponding activity toward the MtM conversion reaction.

These results show that Cu‐omega is a highly dynamic system, with the Cu occupancies and the resulting activity varying significantly under different conditions and temperatures. A schematic representation of the different configurations of Cu observed under all the examined conditions is depicted in Figure 3.

At room temperature, Cu‐omega exhibits a high degree of hydration. This may be seen in Figure S8, which depicts the DFM obtained by subtracting the Cu‐omega structure model from the neutron powder diffraction (NPD) data recorded at room temperature. The differential nuclear densities reveal a significant presence of water in the center of *gme* cages, as well as in the 12 MR. Quantitative EPR analysis on Cu‐omega at room temperature shows that approximately ~91 % of the Cu is present as monomers (Figure S1) in a hydrated state (Figure S3a). No other ordered Cu‐sites are observed via AXRPD. This suggests that the remaining 7 % of the total Cu in the system, invisible via EPR spectroscopy, may be present as dimers or in a disordered configuration.^[51]^

At 230 °C, the methanol yield is relatively low, and decreases slightly over the three consecutive cycles (Figure 1). Very little to no 6 MR Cu is observed at this temperature (Figure 2). Even though more potentially active Cu is seemingly available in the 8 MR than at higher temperatures, the yield stays low. One potential source of this behavior may be the significant amount of water present in the system, as seen in the TGA (Figure S5). The majority of the water molecules are retained in the 12 MR channel at RT (Figure S8). The TGA of Cu‐omega (Figure S5), shows a significant loss of water before 230 °C, which will therefore primarily stem from water desorbing from the 12 MR, while significant levels may remain in the 8 MR and *gme* cages. Comparing the TGA of an unloaded and Cu‐loaded zeolite omega shows that the introduction of Cu will cause the sample to retain more water (Figure S5). When comparing the Cu‐occupancies at 230 °C between oxygen and methane exposure, a significant change in the fractions of Cu(2) and Cu(3) is observed. This suggests that the high levels of water remaining in the zeolite aid the migration of Cu throughout the framework.^[42]^ A portion of this water will be coordinated to the Cu ions themselves, as determined via the LCA in Figure S6. At 230 °C a significant fraction of Cu remains in a hydrated form, causing it to be able to easily migrate throughout the framework,^[42]^ as well as be inactive toward the activation of methane. The LCA (Figure S6) shows that the fraction of hydrated Cu is higher than the fraction of Cu located in the 6 MR of the *gme* cages (Figure 2d). As per the mechanism, two Cu's are necessary for the formation of one methoxy group.^[30]^ If one of the two proximal Cu's necessary for methane activation is in a hydrated state the site may potentially be rendered inactive. Further cycling at 230 °C will introduce more steam during each loop, which due to the low temperature may potentially remain in the system for subsequent isothermal cycles. This may be the source for the slight decrease in yield observed at subsequent cycles performed at 230 °C (Figure 1). While a slight increase in yield from cycles two to four may be seen, the change is very small, and therefore we refrain from speculating on their cause.

Increasing the temperature from 230 °C to 290 °C causes a migration of a fraction of the Cu from an active 8 MR position to an inactive 6 MR position in the *gme* cages. This may be attributed to water in a void of the *gme* cages desorbing, which therefore permits the migration of Cu into the 6 MR of the *gme* cages. A change in the ratio of the two 8 MR Cu sites is also observed. While a significant portion of Cu migrates to an inactive position, the yield increases significantly over consecutive cycles at 290 °C. This may be due to more Cu being able to partake in the MtM reaction after losing its hydration shell. As depicted in Figure S6, almost all Cu will be present in dehydrated form above ~275 °C. Another possible source of this behavior may be that over successive cycles Cu may migrate through the 8 MR *c*‐glide channels to a thermodynamically more stable position in proximity of a further Cu, to form the paired monomers that are active for the MtM conversion. The sufficiently low Si/Al ratio of the material (Si/Al of 4.3) allows for a Cu migration via the 8 MR *c*‐glide channels, thereby enabling migration between different unit cells.[^29^, ^39^] Such a behavior has also been witnessed in the case of Cu‐SSZ‐13 for the MtM conversion, albeit in catalytic operational mode.^[42]^

Prolonged cycling using the HT‐protocol causes a successive deactivation of Cu‐omega until a yield of ~40 μmol g_zeolite_^−1^ is reached (Figure 1). This decrease in yield is associated with an unpairing of the Cu active site, caused by a migration of a significant fraction of Cu from the 8 MR to the inactive position in the 6 MR of the *gme* cage. This is also in agreement with previous observations made on Cu‐exchanged SSZ‐13, where a successive dehydration of the zeolite causes a migration of Cu from the 8 MR to the 6 MR.[^33^, ^41^] However, it has also been suggested that in the case of Cu‐SSZ‐13 such a migration is redox‐driven.^[33]^ Such a redox‐driven migration seems to, if at all, only play a minor role t in the case of Cu‐omega, where a temperature ramp to 450 °C under oxygen has given no indication of a change of oxidation state of Cu.^[5]^ EPR measurements on Cu‐omega after an overnight vacuum treatment at 450 °C further exhibit spectral features associated with the inactive Cu in the 6 MR of the *gme* cages.^[52]^ This demonstrates that these sites retain their oxidation state, and are not reducible under the applied conditions. The significant loss of water from Cu‐omega opens a pathway to a *gme* cage. This significant loss of water is depicted in the TGA of both an unloaded and Cu‐loaded zeolite omega material (Figure S5). In the case of Cu‐omega, the TGA‐MS shows that at a temperature above ~400 °C a significant amount of water will desorb. The migration of one of two proximal monomers will suffice to render them both inactive.

Cycling at 290 °C after using the HT‐activation protocol shows that the observed decrease in yield can be reversed by choosing a more appropriate protocol. The yield of the first cycle post HT‐activation protocol and last cycle pre HT‐activation protocol is very similar, at 154 μmol g_zeolite_^−1^ and 147 μmol g_zeolite_^−1^ respectively. Over successive cycles, more Cu will form proximal monomers until the maximum potential number of pairs under the applied conditions is achieved. This is shown by an initial increase from the first to the second cycle post HT‐activation protocol, followed by a stabilization of the yield over the following cycles (Figure 1). In the case of 290 °C, this corresponds to a yield of ~200 μmol g_zeolite_^−1^. The increased number of paired monomers may to a significant extent be derived from Cu migrating from the inactive position in the 6 MR of the *gme* cages, to where it has migrated during use of the HT activation protocol, back to the 8 MR. The cause of this re‐migration may stem from the constant re‐introduction of steam during each loop. This is observed in Figure S7, which depicts the occupancies of the different Cu‐species under an oxygen atmosphere. At 290 °C, with a previous cycle having been performed at 230 °C, the fraction of Cu(1) is significantly lower than at the lower temperatures of 250 °C and 275 °C. The pace at which this process occurs is reflected in the number of cycles it takes for the yield to stabilize. This stabilization in yield also shows that at 290 °C the Cu prefers to be in close proximity to another Cu, thereby constituting the desired proximal monomer pair responsible for the MtM conversion. However, as the Cu‐usage is only ~0.6 (Figure S9), significant amounts of Cu persist in the 6 MR of the *gme* cage or are present as lone monomers in the 8 MR, therefore remaining inactive for the MtM conversion.

## Conclusion

The presented investigation delineates the source of the divergent activity exhibited by Cu‐omega for the partial oxidation of methane under different conditions. The main reason for this divergence in activity is the migratory behavior that Cu exhibits in zeolite omega. This migration of Cu in zeolite omega is predominantly governed by the degree of hydration, and significantly affects the efficacy of Cu‐omega for the partial oxidation of methane. Water clearly has a more nuanced role than solely being an extraction medium for partial methane oxidation via Cu‐zeolites. These insights are primarily achieved using in situ HR‐AXRPD. The amount of information that may be derived from the performed high‐resolution in situ AXRPD studies underscores its value for unraveling the underlying mechanisms dictating a material's performance. This extends beyond the world of direct MtM conversion and will be true for the examination of materials used in NH_3_‐SCR conversion, among others. Investigating a material's efficacy for a given reaction therefore needs to be performed over a wide parameter space. In the case of MtM via Cu‐zeolites, this does not just include different activation protocols, but a variety of temperatures as well the detailed investigation of each stage of the oxygen looping cycle. The insights gained by this in‐depth examination of Cu‐omega suggest that a re‐evaluation of Cu‐zeolites considered to have low activity for the partial oxidation of methane over a wider parameter space may be warranted.

## Conflict of Interests

The authors declare no conflict of interest.

1

## Supporting information

As a service to our authors and readers, this journal provides supporting information supplied by the authors. Such materials are peer reviewed and may be re‐organized for online delivery, but are not copy‐edited or typeset. Technical support issues arising from supporting information (other than missing files) should be addressed to the authors.

Supporting Information

## Data Availability

The data that support the findings of this study are available from the corresponding authors upon reasonable request.
