# Supplementary material for: Quantifying the Hydration‐Dependent Dynamics of Cu Migration and Activity in Zeolite Omega for the Partial Oxidation of Methane
Source: Angew Chem Int Ed Engl. 2024 Nov 2;63(49):e202407395. doi: 10.1002/anie.202407395 (PMC11586698; doi:10.1002/anie.202407395)
Supplement: Supplementary file 1 — Supporting Information [file ANIE-63-e202407395-s001.pdf]

Angewandte Chemie
Eine Zeitschrift der Gesellschaft Deutscher Chemiker

Supporting Information

Quantifying the Hydration-Dependent Dynamics of Cu Migration and Activity in Zeolite Omega for the Partial Oxidation of Methane

J. Wieser, D. Wardecki, J. W. A. Fischer, M. A. Newton, C. Dejoie, A. J. Knorpp, T. C. Hansen, G. Jeschke, P. Rzepka, J. A. van Bokhoven**

Quantifying the Hydration-Dependent Dynamics of Cu Migration and Activity in Zeolite Omega for the Partial Oxidation of Methane

Johannes Wieser,#,[a] Dariusz Wardecki,#,[b] Jörg W. A. Fischer,[c] Mark A Newton,[a],[d] Catherine Dejoie,[e] Amy J. Knorpp,[a] Thomas C. Hansen,[f] Gunnar Jeschke,[c] Przemyslaw Rzepka,*[a],[d],[g] Jeroen A. van Bokhoven*[a],[g]

[a] Department of Chemistry and Applied Biosciences, Institute for Chemical and Bioengineering, ETH Zurich, 8093 Zürich, Switzerland

[b] Institute of Experimental Physics, Faculty of Physics, University of Warsaw, 02-093 Warsaw, Poland

[c] Department of Chemistry and Applied Biosciences, Institute of Molecular Physical Science, ETH Zurich, 8093 Zürich, Switzerland

[d] Department of Structure and Dynamics in Catalysis, J. Heyrovsky Institute of Physical Chemistry, Dolejškova 2155/3, 182 23 Prague 8, Czech Republic

[e] ID22, European Synchrotron Radiation Facility, 38043 Grenoble, France

[f] Institut Laue-Langevin, 71 Avenue des Martyrs, 38000 Grenoble

[g] Center for Energy and Environmental Sciences, Paul Scherrer Institute (PSI), 5232 Villigen, Switzerland

[#] These authors contributed equally to this work

Supplementary Information

Experimental section

Material properties

Zeolite omega was synthesized in its sodium form using an in-house built rotating oven.[1,2] The synthesis time was 20 days at 110 °C. Post synthesis, the sample was calcined in air at 550 °C for eight hours (ramp rate of 1 K/min).[2] The material was then ion-exchanged with 2 M NH_4NO_3 solution (50 °C, 24 hours). The sample was then ion-exchanged with 0,0025 M $Cu(NO_3)_2$ solution (50 °C, 24 hours) a total of three times. After each ion-exchange the sample was washed with water and ethanol. The final Cu-omega material exhibits a Si/Al of 4.3 and a Cu loading of 4.4 wt %.

Experimental setup

Reactivity studies

The Cu-omega sample was pelletized to 5 tonnes, mortared, and then sieved to 75-125 µm fraction. 5 mg of sample was loaded into a quartz capillary of 2 mm inner diameter and 0,1 mm wall thickness. Quartz wool was placed on either side of the bed. The capillary had previously been glued into a bracket using "Clax flytying Light UV Glue" and exposed to UV light using a "Nailgirls Professional Nail Equipment" setup to ensure proper hardening of the glue.

The setup is the same as used for prior studies on Cu-omega, and a detailed schematic of the reactor system is reported in prior work by Wieser et al.[3] A bracket, into which a capillary is glued, is inserted into a bracket holder. The bracket has female Swagelok fittings attached on both sides that attaches to male Swagelok fittings on the bracket holder. The bracket holder has quick connects for attaching the inlet and outlet gas lines. The bracket holder also hosts a thermocouple attachment, which is inserted into the capillary to be as close as possible to the sample without damaging the bed. Heating elements are inserted above and below the capillary bed. This type of heating system has been tested in previous experiments and has been shown to guarantee isothermal heating of the material bed.[3,4] To avoid moisture, all gases are fed through purification columns. The mass flow controllers (BROOKS Smart Mass Flow) were all set to 20 sccm. One helium line is fed through a stainless-steel saturator filled with water. The temperature of the saturator is held constant at 30 °C. Three Valco Vici 4 port switching valves are used to quickly switch between the various gas atmospheres. All lines that experience any flow containing water, or other condensable products such as the product methanol, are traced to 100 °C to avoid condensation.

Electron Paramagnetic Resonance (EPR) Spectroscopy

EPR experiments were performed using a homebuilt, water-cooled, high-temperature resonator, which was installed into a continuous-wave (cw) EPR spectrometer (Bruker EMX) operating at X-band frequencies (~9.265 GHz).[5] The resonator is heated by a flow of hot N_2 using a home-built temperature controller.

Flow-through cell EPR measurements

The flow-through cell consists of two aligned quartz tubes. Inside the EPR quartz reactor (Wilmad; 4 mm OD) a thinner inner capillary (Qsil: 2.0-2.4 mm OD) is inserted and this assembly is connected to the gas supply system.[6] The material bed (ca. 3 mg) was fixed between two quartz wool plugs in the inner capillary to ensure maximal contact with the reaction gas mixture. The experimental setup consisted of mass flow controllers (Bronkhorst) to control the gas flows. A total flow rate of 8 mL min^{-1} was used for the in situ EPR experiments.

Table 1: Measurement parameters for the X-band continuous-wave EPR measurements.

Experiment	Sweep width [mT]	Modulation frequency [kHz]	Modulation amplitude [mT]	Sweep time [s]	Conversion time [ms]	Time constant [ms]	Power attenuation [dB]
Room temperature measurements	200	100	0.2	140	70	40	20

Quantitative EPR measurements

The quantitative EPR experiments were carried out in a batch reactor consisting only of one quartz tube (Wilmad; 4 mm OD) using about 3 mg of the material. The material was held in place by a quartz wool plug. The measurement parameters for the quantitative X-band cw EPR experiments via a batch reactor are summarized in Table S2. The material was subjected to treatment in a vacuum followed by dosing of 400 mbar oxygen at 200°C prior to the room temperature measurements (activated) the sample was treated again in vacuum to remove the oxygen. The sample was then exposed to 10 mbar methane at 200°C and subsequently cooled down again to room temperature (reacted). All spectra were recorded at room temperature.

Table S2. Measurement parameters for the quantitative X-band cw EPR measurements.

Experiment	Sweep width [mT]	Modulation frequency [kHz]	Modulation amplitude [mT]	Sweep time [s]	Conversion time [ms]	Time constant [ms]	Power attenuation [dB]
Quantitate measurements	200	100	0.2	120	60	40	20

It needs to be noted that quantitative measurements with EPR are associated with an error of about 5-10%.[7] A series of $Cu(pic)_2$:$Zn(pic)_2$ Cu(II) reference samples has been used as a reference.[8] The Cu-MAZ sample and references were measured on the same day without retuning the instrument. Special care has been taken to ensure that the reference and sample occupy the same volume within the quartz tube and that they were measured using quartz tubes with the same specifications.

Combined high-resolution in situ anomalous x-ray powder diffraction (HR-AXRPD) and XAS

BM28

The first attempt of consecutive HR-XRPD and XAS measurements was carried out at BM28 (XMaS, UK CRG Beamline) at the European Synchrotron Radiation Facility (ESRF) in Grenoble, France. The investigation focused on the Cu-omega sample during its thermal activation under flowing oxygen at 170 °C, 250 °C, and 275 °C. The sample was mounted in the same reactor system as reported by Wieser et al.[5], as a packed bed (no more than 5 mm long), secured by small quartz wool plugs, within a quartz capillary with 0.5 mm inner diameter and 0.01 mm wall thickness. A 6 circle Huber system allowed the capillary to be mounted along the center of rotation and in the horizontal axis normal to the x-ray beam.

Data collection involved scanning the Pilatus 300K 2D camera mounted on the first optical rail through 4 to 54 degrees in steps of 0.25 degrees at the x-ray energies used for conventional diffraction data (17.5 keV) and anomalous (resonant) diffraction (selected, for the Cu K-edge, at 8.94 and 8.97 keV). A second rail, holding the ion chamber, was used for XAS in transmission mode. By moving the diffractometer 2Θ-arm, XAS and HR-XRPD measurements were thus rapidly interleaved. XAS measurements were conducted in transmission mode, using two Oken ionization chambers filled with an N_2/Ar mix.

ID22

In order to investigate correlations between MtM conversion and changes in the crystal structure of Cu-omega, AXRPD was also employed at the high-resolution powder diffraction beamline ID-22 at the European Synchrotron Radiation Facility (ESRF).[9] In this case, HR-XRPD data were collected using a 13-crystal multianalyzer stage and an Eiger2 CdTe 2M-W pixel detector.[10] The sample was loaded into a 0.5 mm quartz capillary, and mounted on a dedicated flow cell connected to the gas system of the beamline. Heating was achieved using a hot-air blower. In situ HR-AXRPD measurements for two different isothermal cycles (at 230 °C and 290 °C) were performed. Both experiments followed the same protocol, but one was monitored by HR-XRPD measurements at a wavelength off the resonance (0.3543 Å), and the other by HR-AXRPD with the wavelengths near the Cu K-edge (1.3797 Å and 1.3822 Å). Each isothermal cycle was divided into three steps: i) activation under oxygen atmosphere, ii) reaction with methane, iii) desorption of products. During each step, a series of powder diffraction scans was collected until an equilibrium was reached. This was monitored by seeing no significant changes in diffraction peak intensities between consecutive scans. Finally, the scans measured at equilibrium were integrated for better statistics to yield four datasets. In addition, each isothermal cycle was additionally monitored by mass spectrometry. More information about the data processing, Rietveld refinements, and final parameters is provided in Supporting Information.

To further monitor the material, in situ x-ray absorption spectroscopy at the Cu K-edge, conducted in fluorescence yield mode, was interleaved with the HR-XRPD measurements. XANES data from ID22 were collected using a Vortex 1 mm SDD fluorescence detector. XANES permits the monitoring of the speciation of Cu during the measurement, and thereby may be seen as a tool to validate that the material is reacting with the desired atmosphere. Furthermore, a Linear Combination Analysis (LCA) was performed to monitor the variation of hydration

of the Cu at different temperatures. The initial state of the material (hydrated at RT) is used as a reference for a hydrated system, and the material at 290 °C as a reference for a dehydrated system. This comes with multiple potential sources of error. A more detailed discussion of the performed LCA may be found in a subsequent section of this supplementary information (see below). The Cu K-edge XANES are normalized, and the LCA performed using the Prestopronto software package[11], which allows for batch normalization of large datasets, as well as LCA.

Neutron powder diffraction (NPD)

Constant wavelength neutron powder diffraction (NPD) data were collected on the instrument D1B at the Institut Laue-Langevin (ILL) in Grenoble, France during the experiment 5-22-788.[12] The resulting Difference Fourier map (DFM) is depicted in Figure S8 in the supplementary information. The in situ NPD experiment was performed using a gas stick connected through a stainless-steel capillary to the oxygen gas rig. Prior to the NPD measurements, the Cu-omega sample was immersed in D_2O, dried and packed in aluminum cans with a diameter of 0.8 cm. After mounting the sample onto the gas stick and placing it in the beam, the sample was evacuated under vacuum for 3 h. The sample was then heated to 267 °C under 1 bar oxygen pressure for 8 h. The oxygen atmosphere was replaced several times to remove desorbing water from the system. Each step was monitored by NPD (wavelength of 2.52 Å), performed in a range of 2θ: 3 – 120°. The data acquisition time for each scan was 3 min. The scans with no changes in diffraction peak intensities were averaged for better data statistics. The diffraction pattern of the empty container was measured and subtracted for the sample data.

Analytical

Electron Paramagnetic Resonance Spectroscopy

Prior work on Cu-omega has ruled out the presence of dimeric species when applying the HT-activation protocol.[2] To provide further evidence that a monomeric Cu(II) species is both the predominant species in Cu-omega, and the active site for the partial oxidation of methane under the isothermal looping protocol, quantitative EPR measurements have been performed. Under the applied measurement conditions dimeric Cu-oxo species are expected to be EPR invisible due to their anti-ferromagnetic coupling, while monomeric species are detectable.[8] The results of the quantitative EPR measurements on Cu-omega are depicted in Figure S1.

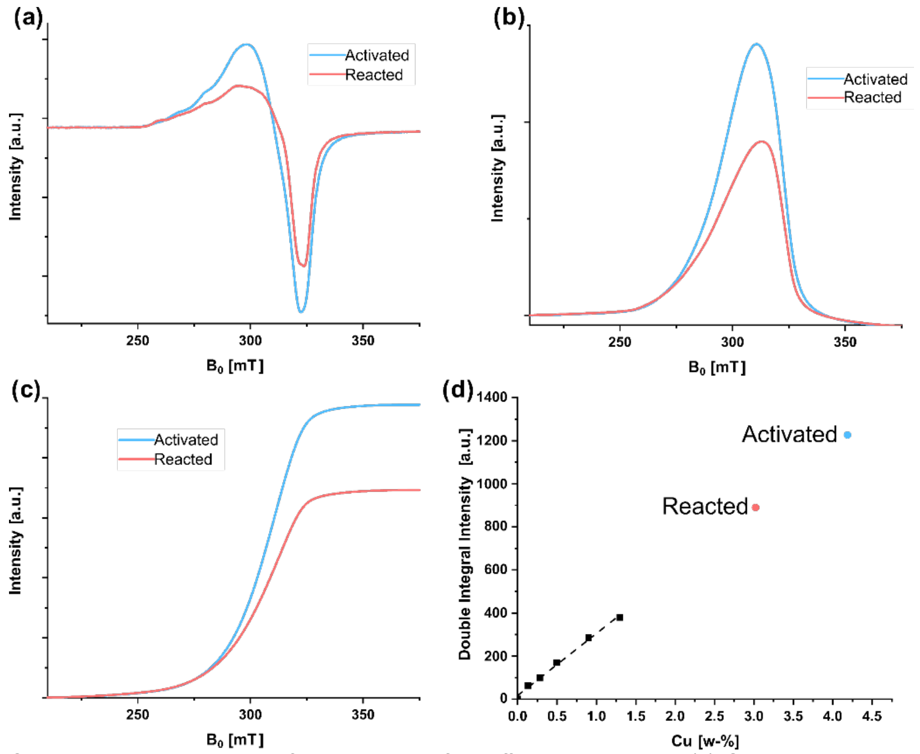

Figure S1: Quantitative EPR analysis of the material after different treatments. (a) Shows the spectra recorded at room temperature, (b) depicts the corresponding first, and (c) the double integrals of Cu-omega after activation under oxygen and reaction with methane. The comparison with the reference samples is shown in (d). The black dots represent the double integral intensity of the reference samples, and the black line is the linear approximation.

The intensity of cw EPR spectra is related, in a proportional manner, to the first derivative of the magnetic susceptibility of the sample at a certain temperature and external magnetic field strength. Because EPR spectra are recorded with the use of field modulation and demodulation, the first derivative of the EPR absorption signal is obtained. Accordingly, the double integral of the EPR spectrum is a direct measure of the total number of spins in the sample.[13] To relate the intensity to an absolute Cu-content of the sample a set of Cu(II) reference samples was employed where the Cu-content is known.[8] These samples have been measured under the exact same conditions as the Cu-omega sample presented in this paper. By integrating the EPR spectra of Cu-omega after activation and reaction (Figure S1b and Figure S1c), as well as the reference samples, the amount of monomeric Cu(II) present in the sample can therefore be determined.

Quantification of the total EPR signal of Cu-omega at room temperature, after the material has been activated under oxygen at 200 °C, indicates about 4.1 wt % of a monomeric species (Figure S1d). This corresponds to ~ 93 % of the total Cu present in the structure, thereby ruling out the presence of large amounts of Cu dimers or species of higher nuclearity.[7] The one-electron reduction of Cu(II) results in a closed shell d^{10} configuration causing Cu(I) to be EPR invisible. Exposure to 10 mbar of methane in the batch reactor leads to a significant drop in EPR intensity of ~ 30 % , demonstrating that monomeric Cu(II) is reducible in the presence of methane.

To shed further light on the dynamic behavior of Cu(II), Cu-omega was investigated via EPR spectroscopy using a flow-through cell. The sample was heated to successively higher temperatures under an oxygen flow of 8 mL min^{-1}. The EPR spectra were subsequently collected at room temperature after having been exposed to temperatures in the range of 200-450 °C under oxygen, and are depicted in Figure S2.

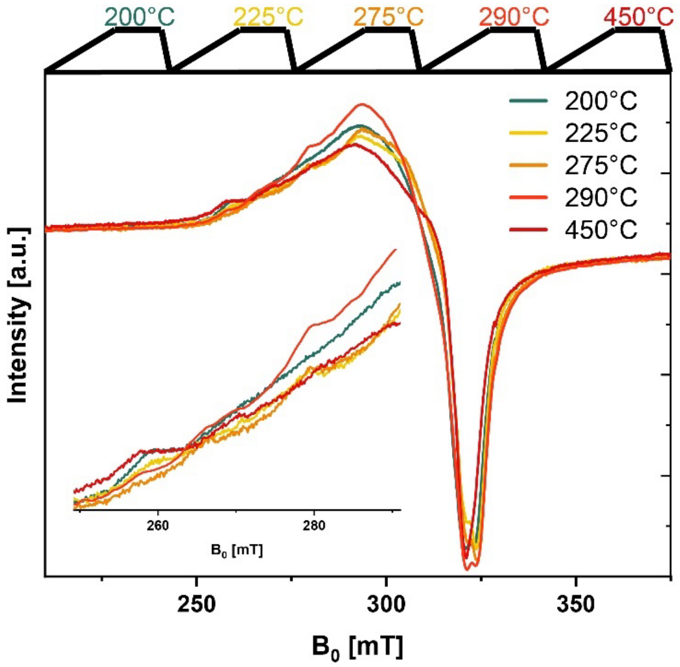

Figure S2: EPR spectra recorded at room temperature after heating the sample in oxygen to the indicated temperatures. The inset highlights the shifts in the g_{II} region of the Cu^{II} spectra. The heating sequence is represented on the top of the figure.

Analysis of the g_{II} region of the spectra indicates a temperature-induced variation in the speciation. The gradual changes observed during heating indicate that the concentration of the Cu(II) species characterized by a g_{II} value of 2.37 is diminishing with increasing temperature This species has been assigned to Cu(II) sites which are partially hydrated (see Figure S2).[14] During the temperature ramp to 450 °C, new spectroscopic signals emerge. The corresponding g values are not clearly distinguishable due to the broad spectral line shape and multiple overlapping species, thereby preventing a quantitative assessment of the different Cu species present in Cu-omega. However, after full dehydration in vacuum and consequential partial reduction of the Cu present a single species dominates the spectrum. The spectra recorded at room temperature prior to heating ramp, as well as after the mentioned vacuum treatment at 450 °C are depicted in Figure S3.

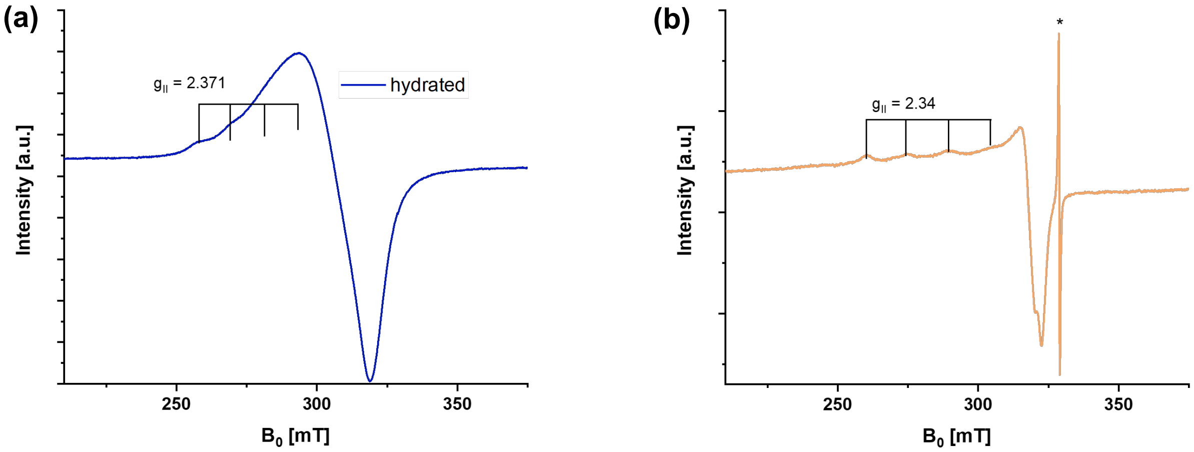

Figure S3: (a) EPR spectra of hydrated Cu-omega. The Cu hyperfine quadruplet at the $g_{||}$ orientation, which is assigned to the hydrated species[14], is highlighted. (b) EPR spectra of Cu-omega treated at 450 °C in vacuum overnight. The spectrum was recorded at room temperature. The asterisk marks the coke radical which forms in the absence of oxygen at high temperatures. The Cu hyperfine quadruplet at the $g_{||}$ orientation belonging to Cu in the 6 MR of the *gme* cages[14] is highlighted.

Figure S3b depicts the spectrum recorded after a vacuum treatment at 450 °C has been performed. The dominating signal after such a treatment is characterized by a g_{II} value of 2.34. This signal has been assigned to a Cu(II) species in the center of a 6 MR of zeolite omega.[14] Prior work on Cu-omega has been able to identify Cu in the 6 MR of the *gme* cages, and determined that this Cu-species is inactive toward the MtM conversion.[2] The gradual change in the g_{II} of the spectra (2.371 to 2.34) with an increase in temperature suggests that the Cu in zeolite omega is able to migrate, and elevated temperature induces a change in coordination of a portion of the Cu atoms.

Mass Spectrometry

Determined Calibration Factors

On-line operando mass spectrometry was performed using the Omnistar GSD 320 Gas Analysis System. The mass spectrometer is attached to the outlet of the reactor system. Mass spectrometry was used for the assessment of gas purity, as well as for the quantification of products.

Quantification via mass spectrometry is based on the internal standard method,[15] in this case using helium gas as the internal standard. Prior work has summarized the exact procedure used to construct the calibration curves, and how these may be used to quantify the products.[3] The calibration factor was determined by flowing helium through a bubbler system filled with methanol. By varying helium line pressure, flow rate, and temperature of the bubbler system the ratio of the two mass fragments examined (mass fragment four for helium, mass fragment 31 for methanol) is varied. A thermocouple is inserted into the methanol to permit precise control of that temperature. Enough time is given for the two observed mass fragments to stabilize. Dimethyl ether (Sigma-Aldrich, 38912-1EA) is calibrated by feeding flows of different compositions into the mass spectrometer and waiting for the ion current of the traced mass fragments (mass fragment 46 for dimethyl ether) to stabilize. The calibration curves derived from these measurements allow for the determination of the calibration factors. These are depicted for methanol in Figure S4a, and for dimethyl ether in Figure S4b.

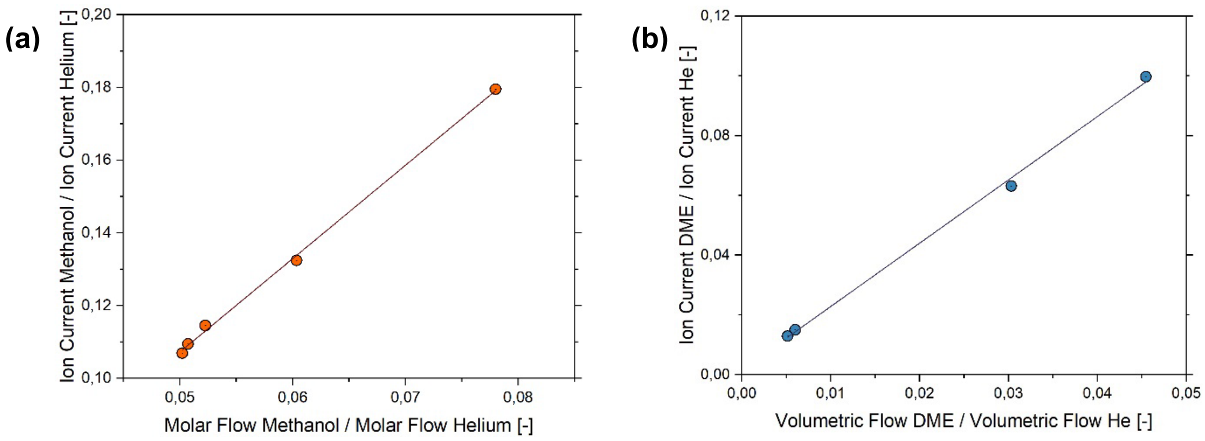

Figure S4: Calibration curves for (a) methanol and (b) dimethyl ether constructed by feeding various compositions of the product and inert gas into the mass spectrometer.

Thermo-Gravimetric Analysis (TGA)

Figure S5 shows the results obtained from the TGA of unloaded and Cu loaded zeolite omega.

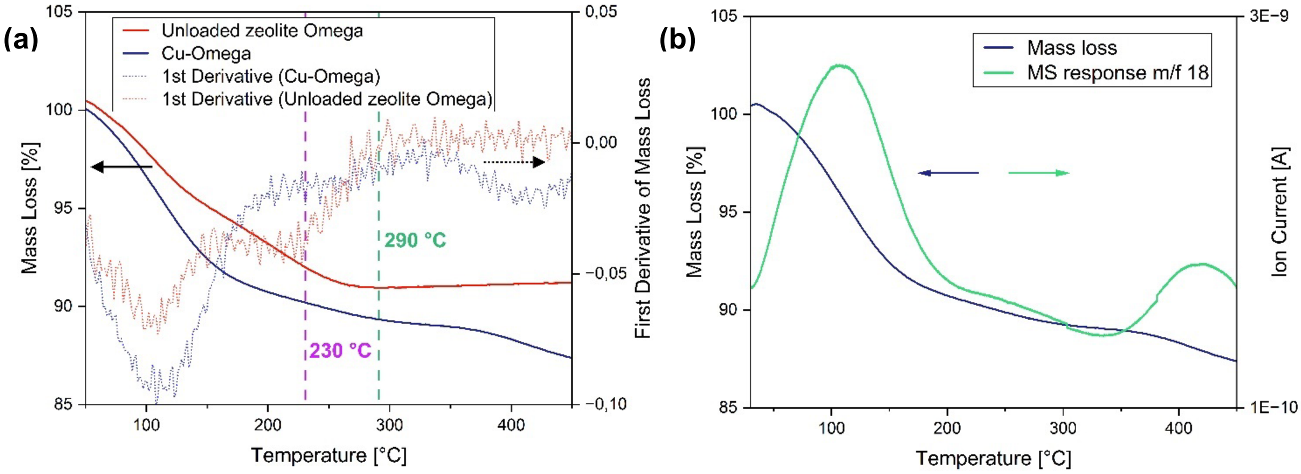

Figure S5: **(a)** Mass loss, as well as the first derivative of mass loss, of unloaded and Cu loaded zeolite omega as a function of temperature. **(b)** Mass loss, as well as mass spectrometer response of mass fragment 18, equated with water, as a function of temperature.

Figure S5a shows that there is a significant difference in the mass loss of unloaded and Cu loaded zeolite omega. A much higher fraction of the initial mass is lost in the case of Cu-omega, when compared to unloaded zeolite omega. Both samples lose a significant amount of their initial mass below ~ 200 °C. Both samples further show significant mass loss up to ~ 290 °C. Unloaded zeolite omega shows no further mass loss past this temperature. Cu-omega however shows a significant further mass loss, starting at ~ 350 °C. As shown in Figure S5(b), in the case of Cu-omega this will be due to the loss of water.

X-ray Absorption Near Edge Structure

Figure S6 depicts a Linear Combination Analysis (LCA) of spectra recorded on ID22 in fluorescence mode and the XMAS beamline in transmission mode, both under an oxygen atmosphere.

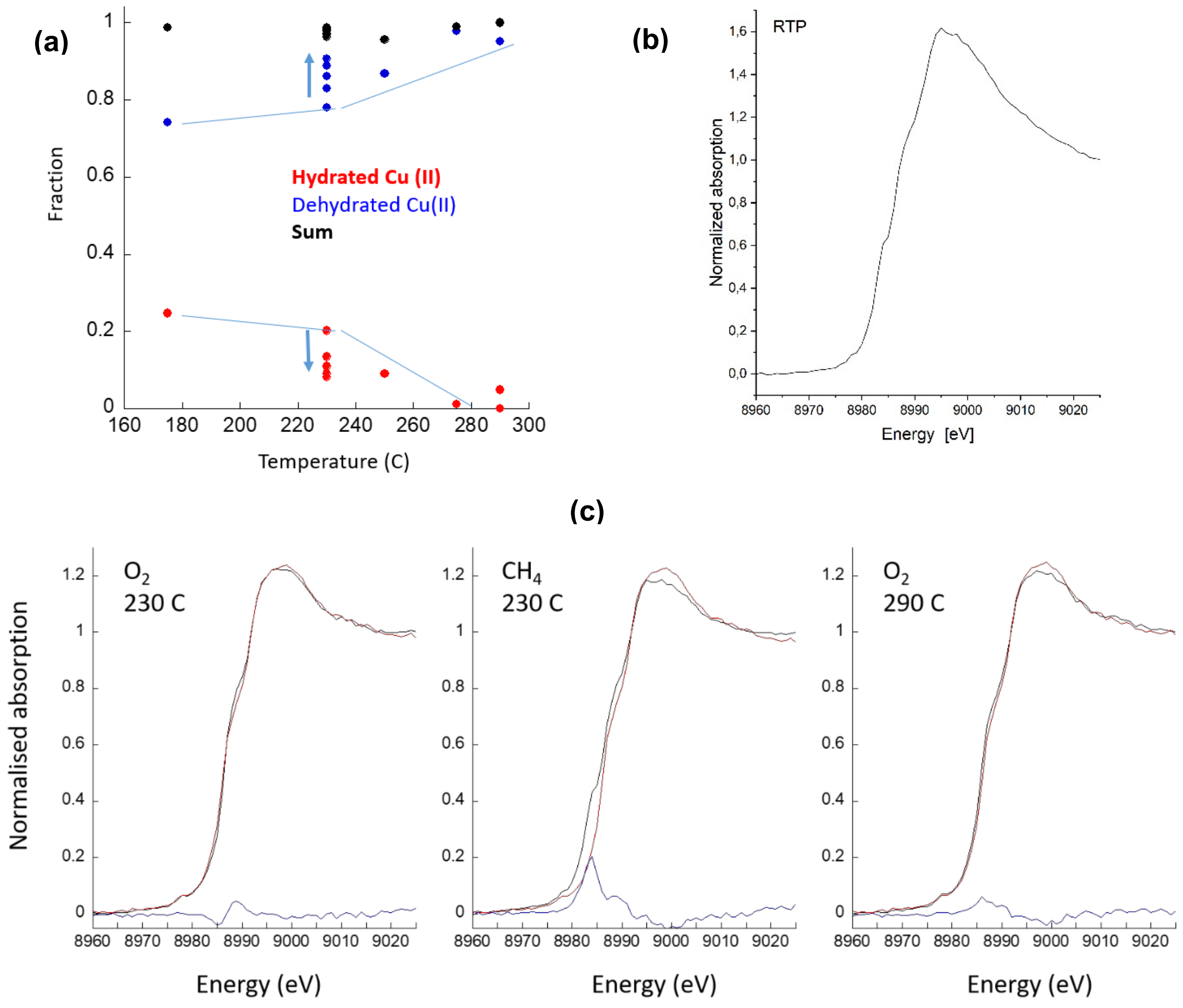

Figure S6: **(a)** LCA-derived Cu speciation from ID22 and XMaS beamlines **(b)** Spectrum recorded at room temperature prior to measurements were conducted. The spectrum is used as the hydrated Cu(II) reference **(c)** Examples of fits and their residuals obtained from LCA of Cu K-edge XANES collected in fluorescence mode on ID22 and for three salient cases as indicated. In each case, the experimental data is given in black, the LCA fit in red, and the residuals in blue. In the case of oxygen at 290 °C, the spectrum is also used as a reference for the LCA.

Figure S6 shows the results of fitting the Cu K-edge fluorescence XANES for hydrated and dehydrated forms of Cu(II). The use of fluorescence detection at ID22 is due to the detection system for PXRD/AXPRD preventing the use of transmission. As such, whilst the interleaving of the XANES measurement with the PXRD/AXRPD is possible, it is compromised in some ways. At the Cu K-edge, self-absorption effects, which diminish the intensities and broaden features in the XANES, are significant.

The LCA data presented in the Figure S6 is obtained using the following assumptions: The spectrum collected at RT under helium and prior to heating constitutes 100 % hydrated Cu(II); the spectrum derived from the sample maintained under oxygen, and held for the longest time at the highest temperature investigated (290 °C) represents the 100% dehydrated Cu(II) case.

The validity of these assumptions is tested in two ways: assessment of the residuals of spectra recorded during the reactive cycling (Figure S6b), and through comparison to XANES data collected during similar experiments at the XMaS beamline (Figure S6a). The XANES collected at XMaS were recorded in transmission mode, and are therefore free of any self-absorption.

From Figure S6 it can be observed that under oxygen at 230 °C and 290 °C the use of only two LCA components results in good fits to the data and acceptable residuals. However, after exposure to methane at 230 °C the fit is worse. However, the nature of the residuals is precisely what we would expect them to be after the samples exposure to methane, namely features corresponding to Cu(I), which are not accounted for by the two component model. At 230 °C product yields (Figure 3) were of the order of 80 µmol $g_{Zeolite}^{-1}$. From the two-electron mechanism of methane activation[16], and assuming 100% selectivity (no over-oxidation) this would suggest that, at most, this would translate to 160 µmol $g_{Zeolite}^{-1}$ Cu(I), and thus ca. 23% of the Cu (see also below, Calculations - Yield) present in the material. At 290 °C under oxygen the low levels of residuals (blue curve) do not correspond to Cu(I). In this case, and with reference to the LCA at 230 °C under methane, where the residuals are at energies of the pre-edge feature associated with Cu(I), the residuals are at energies above said Cu(I) pre-edge feature. As such, no evidence for Cu(I) after activation of Cu-omega at 290 C is indicated.[3]

The second validation of this approach comes from the incorporation of the results derived from XMaS where the Cu K-edge XANES could be collected in transmission and with the relevant internal standards collected and applied. These data add points at 170 °C, 225 °C, 250 °C, and 275 °C (Figure S7), to the points at 230 °C and 290 °C collected on ID22, with the standard for the fully dehydrated Cu(II) case being obtained from activation at 450 °C oxygen as per the conventional HT aerobic activation. It can be observed that these points do not deviate substantially from those collected on ID22, and indeed the overall pattern of behavior thus derived is consistent with the overall form and character of the TGA given above.

In situ AXRPD under oxygen atmosphere

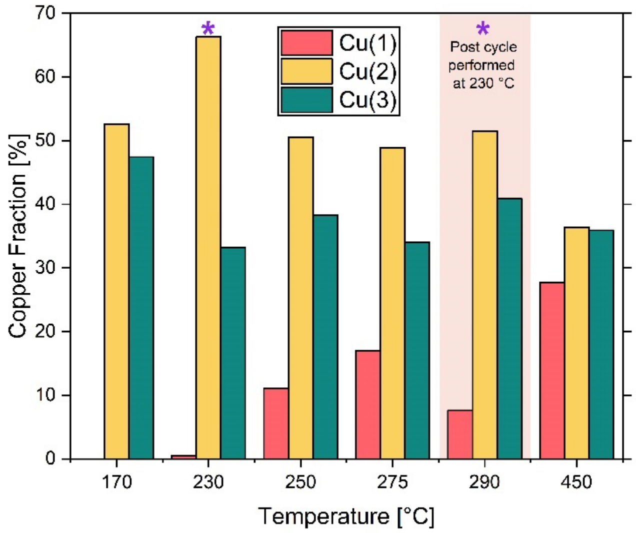

Figure S7: Cu-occupancies of the three possible Cu positions as a percentage of the total Cu population per unit cell at different temperatures under an oxygen atmosphere. The data for 230 °C and 290 °C were collected at ID22 of the ESRF. The data for 170 °C, 250 °C and 275 °C were collected at BM28 of the ESRF. The results obtained for 450 °C were taken from prior work by the authors, and collected at the MS beamline of the SLS.[2]

Figure S7 shows the Cu-occupancies of the possible Cu positions at different temperatures under an oxygen atmosphere. As is depicted in Figure S7, an increase in temperature induces a progressive migration of Cu to the position of Cu(1), which is associated with Cu in the 6 MR of the *gme* cages and Cu that is inactive the MtM conversion. An exception to this are the Cu-occupancies witnessed at 290 °C. In comparison to the other temperatures, a prior cycle has been performed at 230 °C in this case. A whole cycle includes the introduction of steam into the system, which is known to be able to cause a migration of Cu throughout the zeolite framework.[17] The Cu-occupancies at 170 °C, 250 °C and 275 °C instead were recorded during a temperature ramp under oxygen. Therefore, one potential source for the decreased amount of inactive Cu(1) at 290 °C, in comparison to the lower temperatures of 250 °C and 275 °C, may be that the re-introduction of water has caused Cu to migrate to an active position inside the 8 MR of zeolite omega, i.e. from Cu(1) to Cu(2) or Cu(3).

Neutron Powder Diffraction

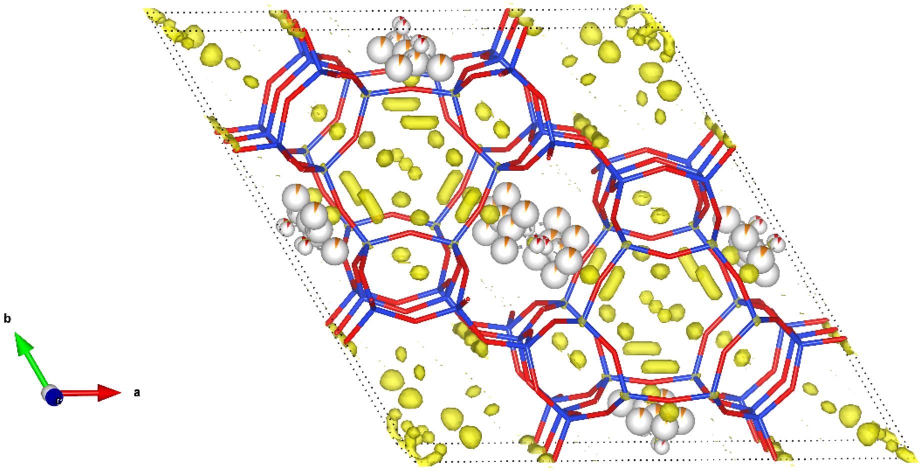

Figure S8: Density Fourier Map obtained via neutron powder diffraction of Cu-omega at room temperature.

Figure S8 depicts the DFM generated by: 1) calculating structure factors (F_{calc}) from the structure model with Cu sites and 2) subtracting obtained (F_{calc}) from observed (F_{obs}) measured from Cu-omega at RT in neutron powder diffraction (NPD) experiment. The density contrast against the framework was enhanced by immersing the material in D_2O before the experiment. The differential nuclear densities reveal a significant presence of water in the center of the *gme* cage and in 12 MR, with smaller amounts present in the 8 MR.

Calculations

Yield

The yield is calculated on a methanol equivalent basis. As dimethyl ether consists of two methoxy groups it is counted as two methanol molecules.[16] The following formula is used for the calculation:

$$Yield = \frac{n_{Product}}{g_{Zeolite}} = \frac{(n_{MeOH}+2\times n_{DME})}{g_{Zeolite}} \quad [\mu mol\ g_{Zeolite}^{-1}] \qquad \text{(Eq.1)}$$

Productivity

The following formula is used for the calculation of the productivity of one whole loop:

$$Productivity = \frac{Yield}{Time}\ [\mu mol\ g_{Zeolite}^{-1}\ h^{-1}] \qquad \text{(Eq. 2)}$$

For both the HT-activation and isothermal looping approach, the timeframes are 30 minutes oxygen exposure, 60 minutes methane exposure and 30 minutes steam exposure. In the case of the HT-activation approach, additional time is needed to heat to and from 450 °C under oxygen. The ramp temperature applied was 10 K min^{-1}. The helium purges in between the reactive gas atmospheres were omitted from the calculations, as no process design for a potential MtM conversion via the oxygen looping approach has yet been suggested, and therefore no predictions can be made on the nature of such parasitic steps in an industrial scale process.[3]

Cu-Usage

As per the suggested mechanism, two Cu's are necessary for the formation of a methanol molecule.[3,16] Therefore, the total yield in methanol equivalents (Eq. 1) is multiplied by two. Equation 3 and equation 4, seen below, are two different methods of calculating the Cu-usage.

$$Cu - Usage = \frac{2 \times Yield_{MeOH\ equiv.}}{Total\ Cu} \qquad \text{(Eq. 3)}$$

Equation 3 represents the Cu-usage as a function of the total amount of Cu present in the system. This calculation will include all unpaired copper monomers in the 8 MR, as well as the isolated inactive Cu(1) in the *gme* cages. To show the potential maximum of active Cu, and calculate the Cu-usage from this, equation 4 is used:

$$Cu - Usage_{Potential\ Active\ Cu} = \frac{2 \times Yield_{MeOH\ equiv.}}{2 \times Cu(1)} \qquad \text{(Eq. 4)}$$

Equation 4 represents the Cu-usage as a function of the maximum potential number of paired monomers at the examined temperature. Both Cu(2) and Cu(3) are necessary to form the active site for the MtM conversion, however Figure 2d shows that a disparity between the two positions exist in the 8 MR, and therefore not all will be active. Additionally, isolated inactive Cu(1) will not contribute to the reaction in any way. For this calculation therefore,

inactive Cu(1) as well as unpaired Cu in the 8 MR is not included in the calculation. As the Cu-occupancy of the Cu(3) position is always lower than that of Cu(2) (Figure 2d), the maximum amount of active sites in the 8 MR the total amount of Cu(3) in the system is multiplied by two, due to two proximal Cu's being necessary for the MtM conversion. The different results for both definitions of Cu-usage are depicted in Figure S9.

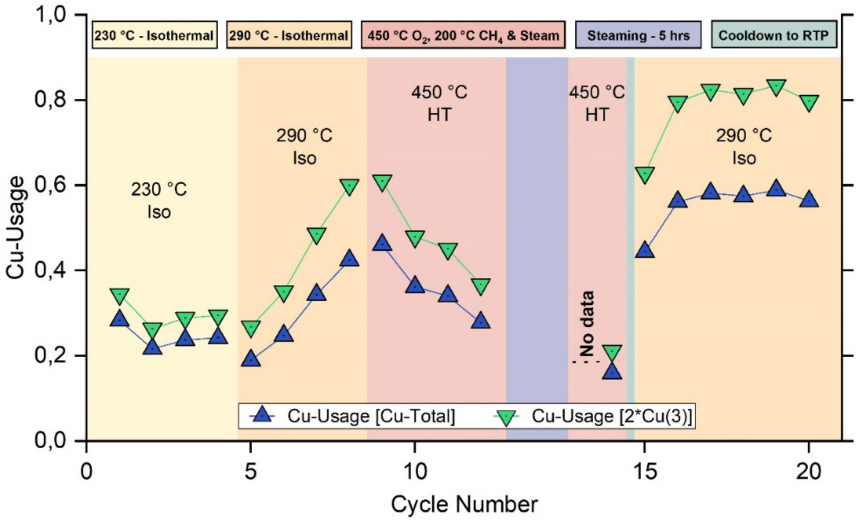

Figure S9: The Cu-usage calculated for both definitions (Eq. 3 and Eq. 4)

As may be seen in Figure S9, even for the definition of Cu-usage of equation 4, not all Cu is being utilized. This holds true even for later cycles at 290 °C, where the yield significantly surpasses that of earlier cycles. This suggests that a significant portion of the Cu present in the 8 MR (Cu(2) and Cu(3), is still inactive toward the MtM conversion.

Cu-Occupancy

The Cu-occupancies, in percent, for the three distinguishable Cu-species, namely Cu(1) Cu(2) and Cu(3) were calculated for different temperatures and atmospheres. They were calculated using the occupancies derived from the Rietveld analysis of the AXRPD data multiplied by the symmetry-related multiplicity of each site and normalized against the total Cu population following equation 6:

$$Cu_x = \frac{occCu_x \times M_x}{occCu_x \times M_x + occCu_y \times M_y + occCu_z \times M_z} \times 100\% \qquad \text{(Eq. 6)}$$

Error of total Cu present in the system

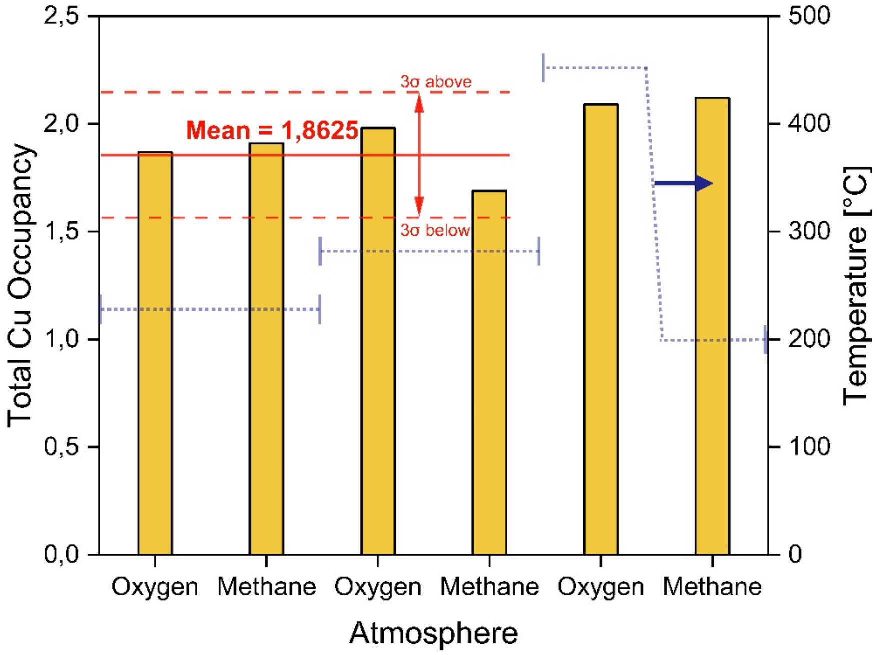

Figure S10: Total Cu-occupancy of Cu-omega under all examined conditions. Data for 230 °C and 290 °C using the isothermal protocol were collected in situ at ID22 of the ESRF, while data for the 450 °C high temperature protocol were collected ex-situ at the MS beamline of the SLS by Knorpp et al.[2] The mean, as well as the three-sigma standard deviation of the Cu present in the system in the case of data collected at ID22 is depicted.

Rietveld analysis of AXRPD data

The Rietveld analysis of the diffraction data was performed using the TOPAS-Academic program. For each dataset, the HR-XRPD and AXRPD patterns were analyzed as a multiple refinement with a common structure model. The initial models of the crystal structures for the activated (Cu_O2_230C, Cu_O2_290C) and reacted (Cu_CH4_230C, Cu_CH4_290C) samples were taken from Knorpp et al.[2] The peak shape in the diffraction data was modeled as a 2θ dependent convolution of the Lorentzian and Gaussian functions with an additional asymmetry. An anisotropic peak broadening was added using spherical harmonics. The background was modeled as a linear interpolation between selected points. The crystal structure for all datasets was refined in the hexagonal system with space group *P*63/*mmc.* The final lattice parameters are summarized in Table S2.

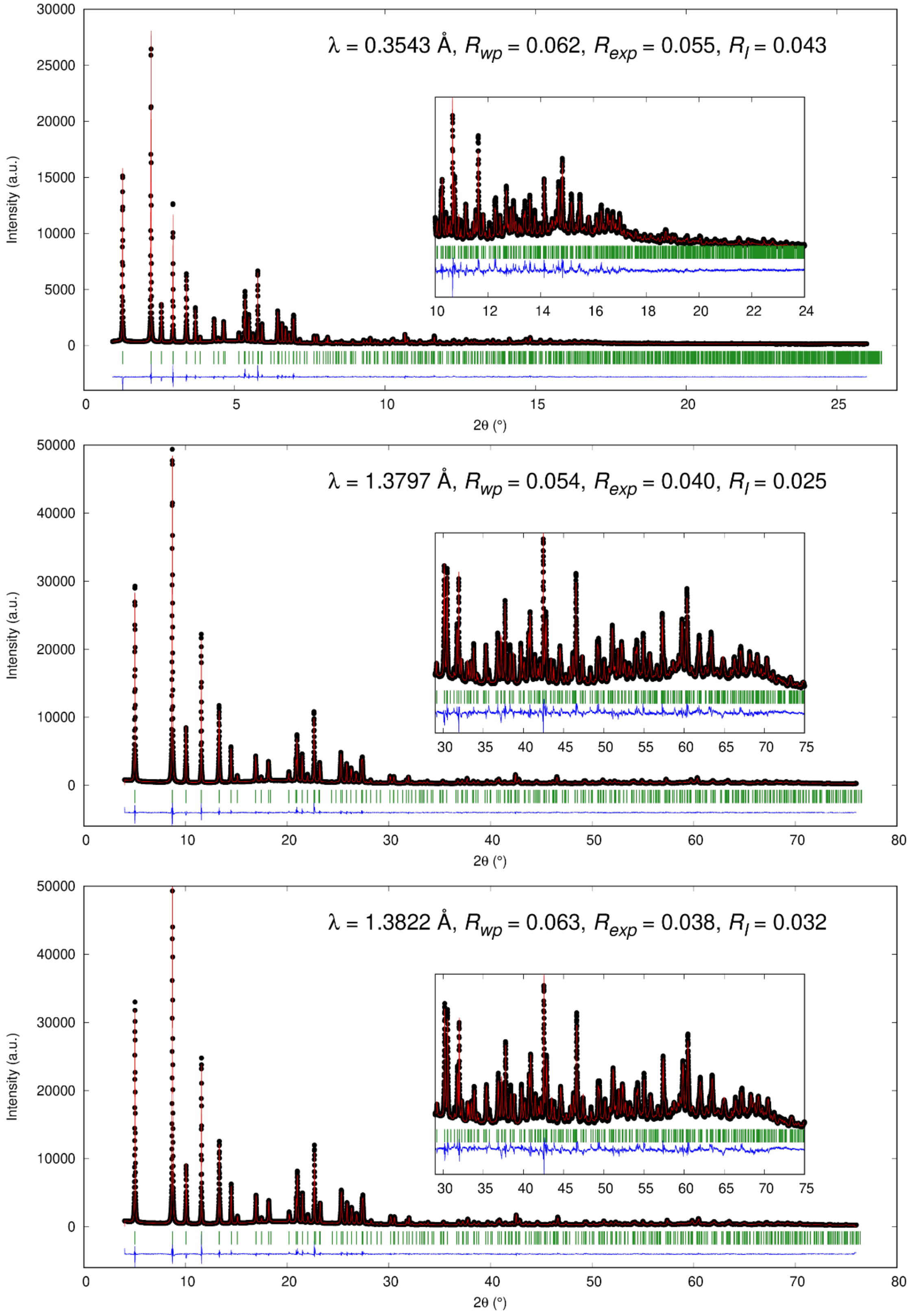

Figure S11: Profile fits (observed: black, calculated: red, difference: blue) for the Rietveld refinement of activated Cu-omega (Cu_O2_230C).

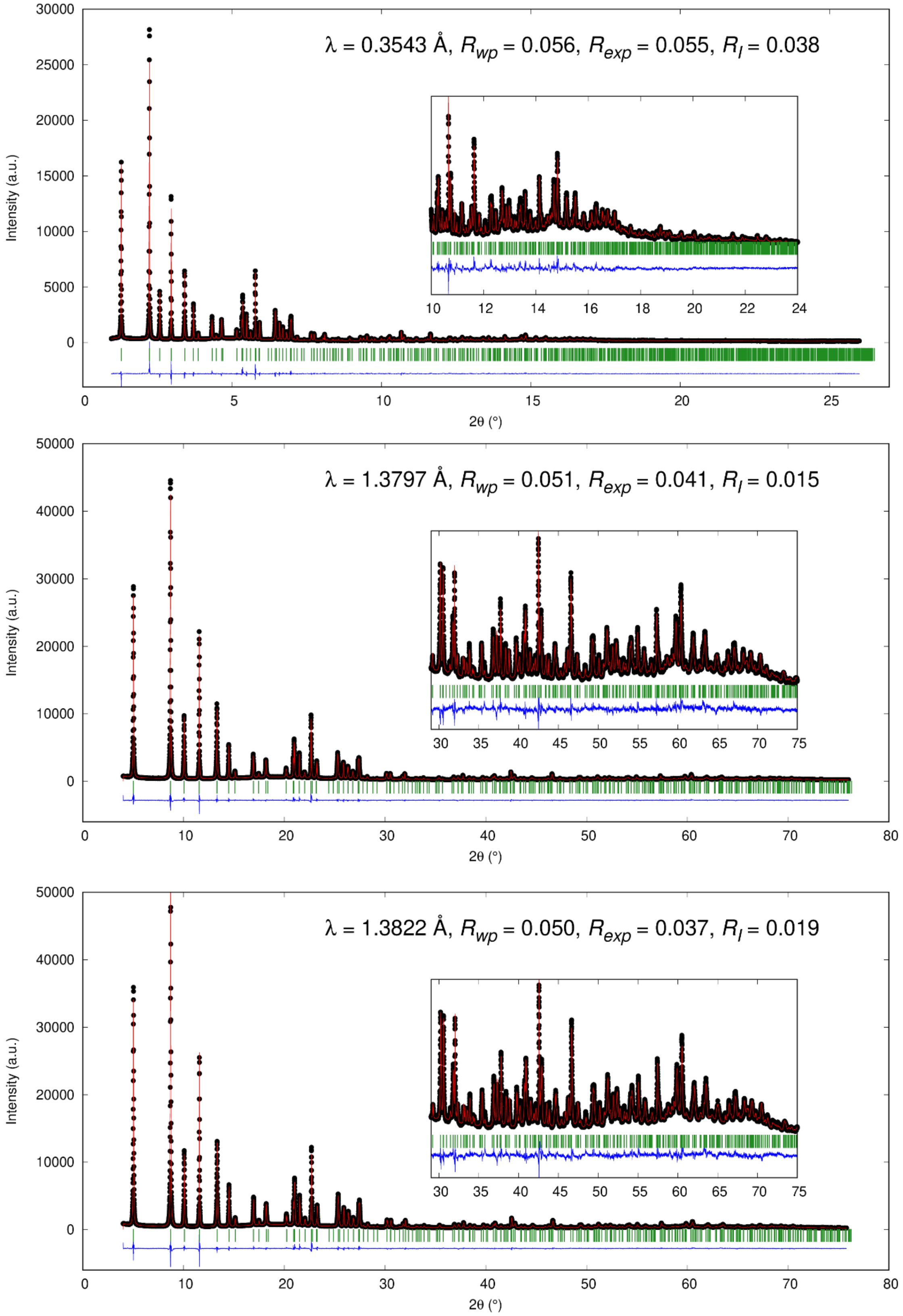

Figure S12: Profile fits (observed: black, calculated: red, difference: blue) for the Rietveld refinement of activated Cu-omega (Cu_O2_290C).

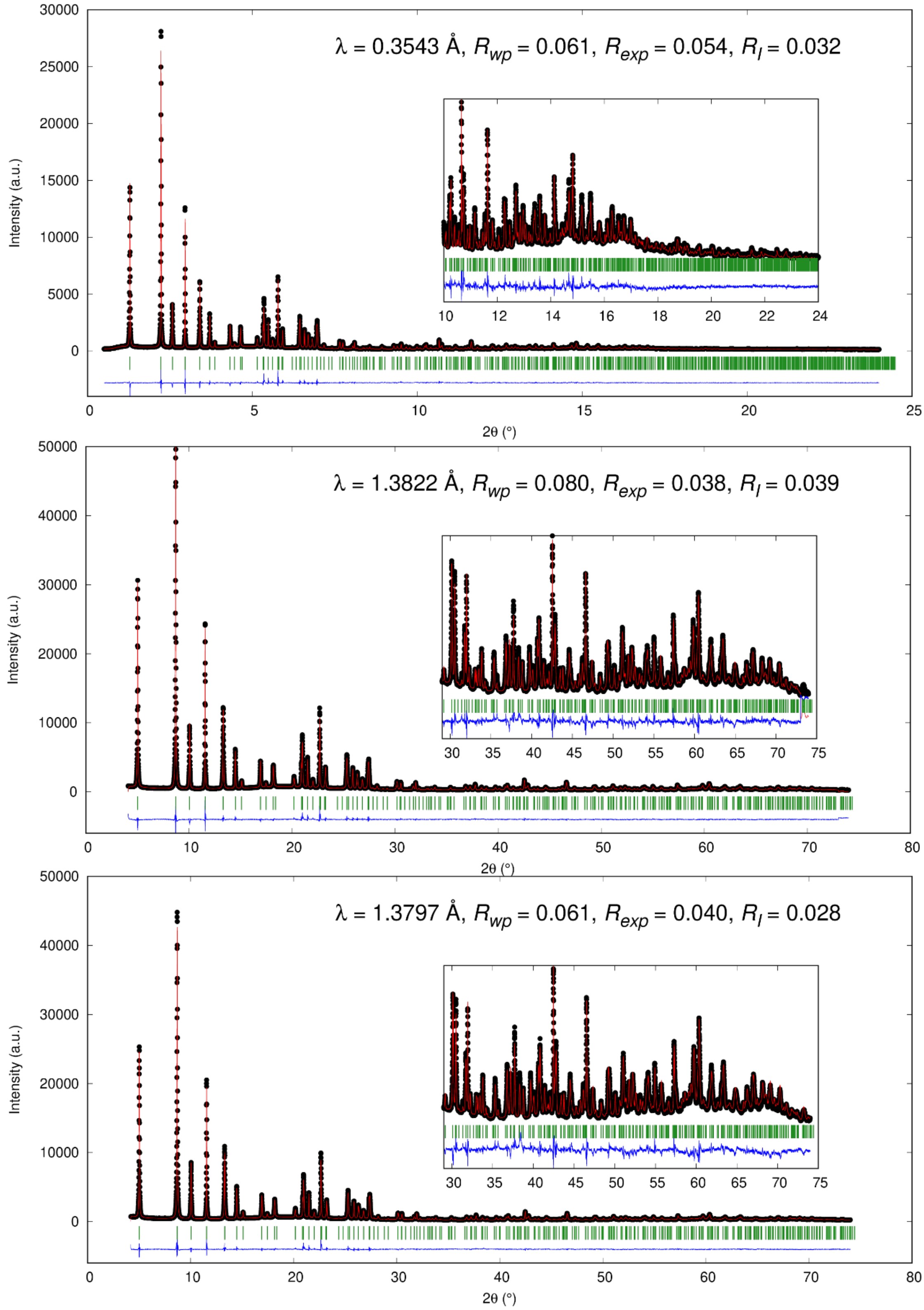

Figure S13: Profile fits (observed: black, calculated: red, difference: blue) for the Rietveld refinement of reacted Cu-omega (Cu_CH4_230C).

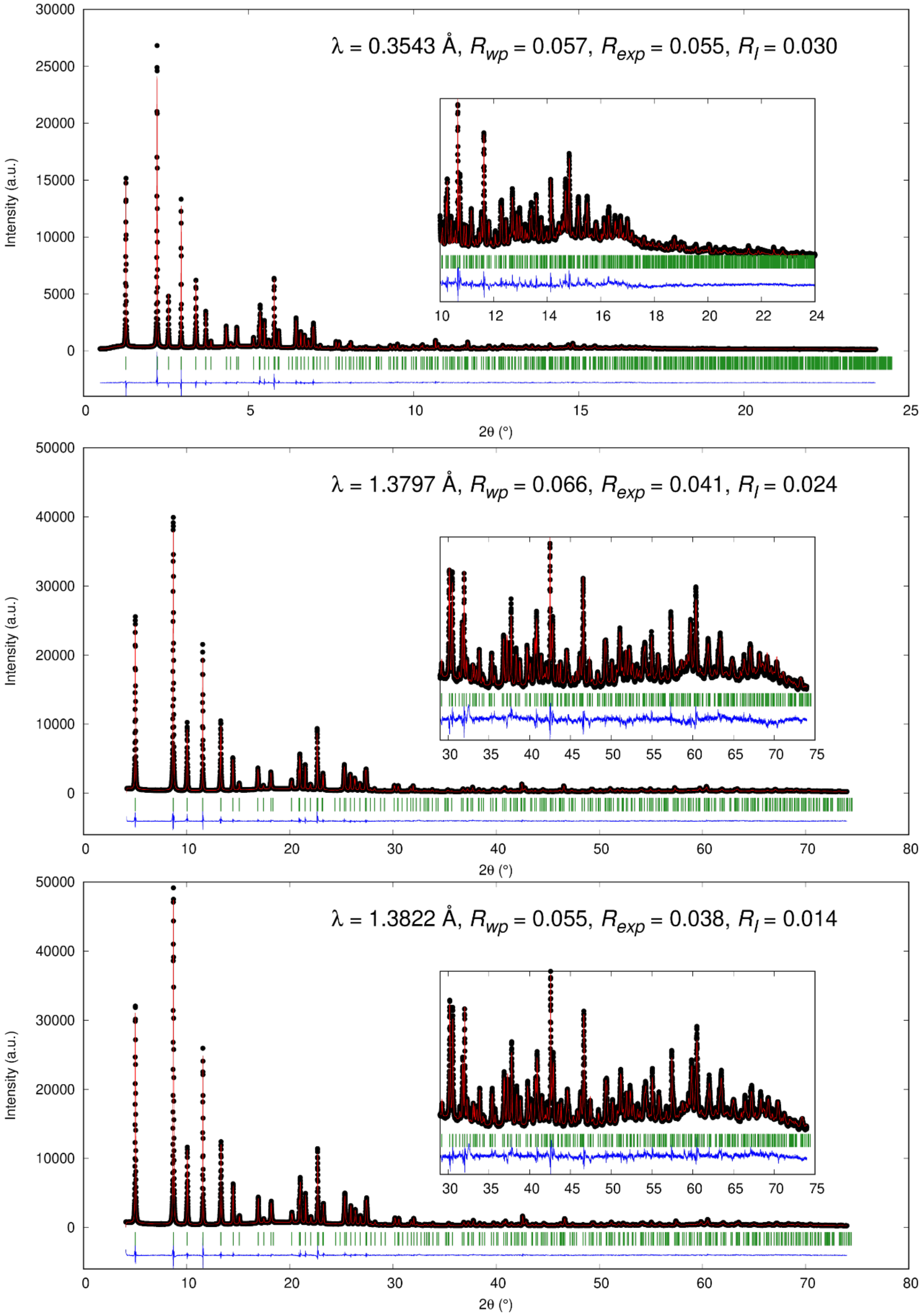

Figure S14: Profile fits (observed: black, calculated: red, difference: blue) for the Rietveld refinement of reacted Cu-omega (Cu_CH4_290C).

Table S3: Crystallographic data

Wavelength independent data				
	**Cu_O2_230C**	**Cu_O2_290C**	**Cu_CH4_230C**	**Cu_CH4_290C**
**Chemical comp.**	$|Cu_{1.9}O_{1.9}|[Si_{29.5}Al_{6.5}O_{72}]$	$|Cu_{2.0}O_{1.5}|[Si_{29.5}Al_{6.5}O_{72}]$	$|Cu_{1.9}O_{1.9}(CH_3)_{0.3}|[Si_{29.55}Al_{6.5}O_{72}]$	$|Cu_{1.7}O_{1.5}(CH_3)_{0.5}|[Si_{29.55}Al_{6.5}O_{72}]$
**Space group**	$P6_3/mmc$	$P6_3/mmc$	$P6_3/mmc$	$P6_3/mmc$
a **(Å)**	18.1491(2)	18.1387(2)	18.1522(2)	18.1436(2)
c **(Å)**	7.5979(1)	7.5984(1)	7.5975(1)	7.5968(1)
**Density (g/cm^3)**	1.771(2)	1.773(1)	1.769(2)	1.766(2)
**Geom. restrains**	4	4	4	4
**Independent param.**	64	63	65	63

Wavelength dependent data				
	**Cu_O2_230C**	**Cu_O2_290C**	**Cu_CH4_230C**	**Cu_CH4_290C**
**Wavelength (Å)**	0.3543	0.3543	0.3543	0.3543
**2 θ range (°)**	0.95 - 26.0	0.95 - 26.0	0.6 - 24.0	0.6 - 24.0

**Profile param.**	10	10	10	10
R_{wp}	0.062	0.056	0.061	0.057
R_{exp}	0.055	0.055	0.054	0.055
R_I	0.043	0.038	0.032	0.030
**Observations**	12525	12525	11700	11700
**Reflections**	878	877	716	716

**Wavelength (Å)**	1.3797	1.3797	1.3797	1.3797
**2 θ range (°)**	4.0 - 76.0	4.0 - 76.0	4.1 - 74.0	4.1 - 74.0
**Profile param.**	10	10	10	10
R_{wp}	0.054	0.051	0.061	0.066
R_{exp}	0.040	0.041	0.040	0.041
R_I	0.025	0.015	0.028	0.024
**Observations**	14400	14400	13980	13980

**Reflections**	328	326	310	310
f'	-9.2(2)	-9.8(2)	-10.2(2)	-10.4(2)
f''	2.6	3.8	3.8	3.8
**Wavelength (Å)**	1.3822	1.3822	1.3822	1.3822
**2 θ range (°)**	4.0 - 76.0	4.0 - 75.8	4.1 - 74.0	4.2 - 74.0
**Profile param.**	10	10	10	10
R_{wp}	0.063	0.050	0.080	0.055
R_{exp}	0.038	0.037	0.038	0.038
R_{I}	0.032	0.019	0.039	0.014
**Observations**	14400	14360	13980	13960
**Reflections**	326	325	308	308
f'	-7.0(2)	-8.0(1)	-9.1(2)	-9.3(2)
f''	0.49	0.49	0.49	0.49

Table S4: Selected interatomic distances (Å) and angles (°) for the samples activated in oxygen.

	Cu_O2_230C	Cu_O2_290C
Si(1) - O(1)	1.64(1)	1.63(1)
Si(1) - O(2)	1.63(1)	1.63(1)
Si(1) - O(3)	1.64(1)	1.63(1)
Si(1) - O(4)	1.63(1)	1.63(1)
Si(2) - O(2)	1.66(1)	1.64(1)
Si(2) - O(5)	1.61(1)	1.62(1)
Si(2) - O(6)	1.62(1)	1.59(1)
O(1) - Si(1) - O(2)	109.8(1)	109.5(1)
O(1) - Si(1) - O(3)	107.1(1)	108.0(1)
O(1) - Si(1) - O(4)	108.7(1)	109.1(1)
O(2) - Si(1) - O(3)	111.7(1)	111.7(1)
O(2) - Si(1) - O(4)	105.5(1)	106.1(1)
O(3) - Si(1) - O(4)	114.0(1)	112.4(1)

O(2) - Si(2) - O(2)	110.7(1)	110.7(1)
O(2) - Si(2) - O(5)	110.6(1)	110.1(1)
O(2) - Si(2) - O(6)	111.0(1)	106.9(1)
Cu(1) - O(5)	-	2.32(1)
Cu(1) - O(61)	-	1.89(1)
Cu(2) - O(2)	2.35(1)	2.26(1)
Cu(2) - O(6)	2.34(3)	2.15(1)
Cu(2) - O(10)	1.82(3)	1.82(1)
Cu(3) - O(4)	2.33(1)	2.06(1)
Cu(3) - O(10)	1.86(2)	1.82(1)
Cu(2) - Cu(3)	3.49(1)	3.50(1)

O(10)_1 – O(10)_2	3.15(7)	2.30(4)

Table S5: Selected interatomic distances (Å) and angles (°) for the samples measured in CH_4

	Cu_CH4_230C	Cu_CH4_290C
Si(1) - O(1)	1.63(1)	1.64(1)
Si(1) - O(2)	1.61(1)	1.62(1)
Si(1) - O(3)	1.64(1)	1.63(1)
Si(1) - O(4)	1.63(1)	1.64(1)
Si(2) - O(2) Si1 - 0005	1.64(1)	1.63(1)
Si(2) - O(5) Si1 - 0008	1.64(1)	1.63(1)
Si(2) - O(6) Si1 - 0007	1.60(1)	1.61(1)
O(1) - Si(1) - O(2) 005	110.6(1)	110.2(1)
O(1) - Si(1) - O(3) 004	105.5(1)	107.1(1)
O(1) - Si(1) - O(4) 003	109.5(1)	109.3(1)
O(2) - Si(1) - O(3) 004	111.2(1)	112.0(1)
O(2) - Si(1) - O(4) 003	106.0(1)	106.2(1)
O(3) - Si(1) - O(4) 003	114.1(1)	111.9(1)

O(2) - Si(2) - O(2) 005	110.3(1)	110.2(1)
O(2) - Si(2) - O(5) 008	109.0(1)	110.6(1)
O(2) - Si(2) - O(6) 007	107.5(1)	106.8(1)
Cu(1) - O(5)	2.24(1)	2.29(1)
Cu(1) - O(61)	2.20(2)	1.91(1)
Cu(2) - O(2) 005	2.29(1)	2.30(1)
Cu(2) - O(6) 007	2.06(1)	2.19(1)
Cu(2) - O(10)	1.96(1)	1.86(1)
Cu(3) - O(2)	2.29(1)	2.28(1)
Cu(3) - O(10)	1.93(1)	1.83(1)
O(10) - C	1.50(7)	1.44(5)

CIF file the final structure of Cu_MAZ_O2_230C

```
data_topas_cif_out

_chemical_name_mineral ?35keV?

_cell_length_a 18.14914(19)

_cell_length_b 18.14914(19)

_cell_length_c 7.59783(6)

_cell_angle_alpha 90

_cell_angle_beta 90

_cell_angle_gamma 120

_cell_volume 2167.37(5)

_symmetry_space_group_name_H-M P63/mmc

loop_

_symmetry_equiv_pos_as_xyz

 'x, y, z '

 '-x, -x+y, z+1/2 '

 '-x, -y, -z '

 '-x, -y, z+1/2 '

 '-x+y, -x, -z+1/2 '

 '-x+y, -x, z '

 '-x+y, y, -z+1/2 '

 '-x+y, y, z '

 '-y, -x, -z+1/2 '

 '-y, -x, z '
```

```
 '-y, x-y, -z+1/2 '
 '-y, x-y, z '
 'y, -x+y, -z '
 'y, -x+y, z+1/2 '
 'y, x, -z '
 'y, x, z+1/2 '
 'x-y, -y, -z '
 'x-y, -y, z+1/2 '
 'x-y, x, -z '
 'x-y, x, z+1/2 '
 'x, y, -z+1/2 '
 '-x, -x+y, -z '
 'x, x-y, -z+1/2 '
 'x, x-y, z '
loop_
_atom_site_label
_atom_site_type_symbol
_atom_site_symmetry_multiplicity
_atom_site_fract_x
_atom_site_fract_y
_atom_site_fract_z
_atom_site_occupancy
_atom_site_B_iso_or_equiv
```

```
Si(1) Si  24 0.73761(5) 0.64401(5) 0.54757(9) 1 2.355(12)

Si(2) Si  12 0.66782(8) 0.50730(8) 0.25 1 2.355(12)

O(1) O  12 0.72403(12) 0.72403(12) 0.5 1 3.03(2)

O(2) O  24 0.67404(10) 0.56194(8) 0.42904(17) 1 3.03(2)

O(3) O  12 0.83727(6) 1.67454(13) 0.5089(2) 1 3.03(2)

O(4) O  12 0.70866(13) 0.61587(14) 0.75 1 3.03(2)

O(5) O   6 0.74103(11) 0.4821(2) 0.25 1 3.03(2)

O(6) O   6 0.5714(18) 0.4286(18) 0.25 0.57(17) 3.03(2)

O(61) O   6 0.583(2) 0.417(2) 0.25 0.43(17) 3.03(2)

Cu(1) Cu+2   6 0.65(9) 0.32(4) 0.25 0.0012(9) 5.0(3)

Cu(2) Cu+2  12 0.4661(2) 0.5339(2) 0.4845(11) 0.1035(12) 5.0(3)

Cu(3) Cu+2  12 0.4110(9) 0.5194(10) 0.25 0.0519(8) 5.0(3)

O(10) O-2  12 0.5004(4) 0.4996(4) 0.207(4) 0.157(2) 5.0(3)
```

CIF file of the final structure of Cu_MAZ_O2_290C

```
data_topas_cif_out

_chemical_name_mineral ?35keV?

_cell_length_a 18.13876(17)

_cell_length_b 18.13876(17)

_cell_length_c 7.59844(5)

_cell_angle_alpha 90
```

```
_cell_angle_beta 90

_cell_angle_gamma 120

_cell_volume 2165.06(4)

_symmetry_space_group_name_H-M P63/mmc

loop_

        _symmetry_equiv_pos_as_xyz

         'x, y, z '

         '-x, -x+y, z+1/2 '

         '-x, -y, -z '

         '-x, -y, z+1/2 '

         '-x+y, -x, -z+1/2 '

         '-x+y, -x, z '

         '-x+y, y, -z+1/2 '

         '-x+y, y, z '

         '-y, -x, -z+1/2 '

         '-y, -x, z '

         '-y, x-y, -z+1/2 '

         '-y, x-y, z '

         'y, -x+y, -z '

         'y, -x+y, z+1/2 '
```

```
    'y, x, -z '
    'y, x, z+1/2 '
    'x-y, -y, -z '
    'x-y, -y, z+1/2 '
    'x-y, x, -z '
    'x-y, x, z+1/2 '
    'x, y, -z+1/2 '
    '-x, -x+y, -z '
    'x, x-y, -z+1/2 '
    'x, x-y, z '
loop_
_atom_site_label
_atom_site_type_symbol
_atom_site_symmetry_multiplicity
_atom_site_fract_x
_atom_site_fract_y
_atom_site_fract_z
_atom_site_occupancy
_atom_site_B_iso_or_equiv
Si(1) Si  24 0.73747(4) 0.64348(4) 0.54648(8) 1 2.319(10)
```

```
Si(2) Si  12 0.66722(7) 0.50680(7) 0.25 1 2.319(10)

O(1) O  12 0.72192(11) 0.72192(11) 0.5 1 3.31(2)

O(2) O  24 0.67490(9) 0.56138(8) 0.42702(15) 1 3.31(2)

O(3) O  12 0.83736(6) 1.67472(12) 0.5100(2) 1 3.31(2)

O(4) O  12 0.71011(12) 0.61492(13) 0.75 1 3.31(2)

O(5) O   6 0.74041(10) 0.48082(19) 0.25 1 3.31(2)

O(6) O   6 0.57430(19) 0.42570(19) 0.25 0.889(7) 3.31(2)

O(61) O   6 0.6067(15) 0.3933(15) 0.25 0.111(7) 3.31(2)

Cu(1) Cu+2   2 0.6666667 0.3333333 0.25 0.074(2) 5.0(3)

Cu(2) Cu+2  12 0.4556(3) 0.5444(3) 0.4969(11) 0.0885(10) 5.0(3)

Cu(3) Cu+2  12 0.4068(7) 0.4943(6) 0.25 0.0704(8) 5.0(3)

O(10) O-2  12 0.5007(6) 0.4993(6) 0.152(2) 0.116(2) 5.0(3)
```

CIF file of the final structure of Cu_MAZ_CH4_230C

```
data_topas_cif_out

_chemical_name_mineral ?35keV?

_cell_length_a 18.1520(2)

_cell_length_b 18.1520(2)

_cell_length_c 7.59739(7)

_cell_angle_alpha 90

_cell_angle_beta 90

_cell_angle_gamma 120

_cell_volume 2167.93(6)

_symmetry_space_group_name_H-M P63/mmc

loop_

_symmetry_equiv_pos_as_xyz

 'x, y, z '

 '-x, -x+y, z+1/2 '

 '-x, -y, -z '

 '-x, -y, z+1/2 '

 '-x+y, -x, -z+1/2 '

 '-x+y, -x, z '

 '-x+y, y, -z+1/2 '
```

```
 '-x+y, y, z '
 '-y, -x, -z+1/2 '
 '-y, -x, z '
 '-y, x-y, -z+1/2 '
 '-y, x-y, z '
 'y, -x+y, -z '
 'y, -x+y, z+1/2 '
 'y, x, -z '
 'y, x, z+1/2 '
 'x-y, -y, -z '
 'x-y, -y, z+1/2 '
 'x-y, x, -z '
 'x-y, x, z+1/2 '
 'x, y, -z+1/2 '
 '-x, -x+y, -z '
 'x, x-y, -z+1/2 '
 'x, x-y, z '
loop_
_atom_site_label
_atom_site_type_symbol
```

```
_atom_site_symmetry_multiplicity

_atom_site_fract_x

_atom_site_fract_y

_atom_site_fract_z

_atom_site_occupancy

_atom_site_B_iso_or_equiv

Si(1) Si  24 0.73762(6) 0.64387(5) 0.54756(11) 1 1.927(14)

Si(2) Si  12 0.66669(10) 0.50733(9) 0.25 1 1.927(14)

O(1) O  12 0.72466(14) 0.72466(14) 0.5 1 2.55(3)

O(2) O  24 0.67746(12) 0.56348(10) 0.4270(2) 1 2.55(3)

O(3) O  12 0.83848(8) 1.67695(15) 0.5100(3) 1 2.55(3)

O(4) O  12 0.70815(16) 0.61420(16) 0.75 1 2.55(3)

O(5) O   6 0.74005(12) 0.4801(2) 0.25 1 2.55(3)

O(6) O   6 0.5720(4) 0.4280(4) 0.25 0.848(17) 2.55(3)

O(61) O   6 0.598(2) 0.402(2) 0.25 0.152(17) 2.55(3)

Cu(1) Cu+1   6 0.68(3) 0.339(14) 0.25 0.0089(12) 4.5(3)

Cu(2) Cu+1  12 0.4565(5) 0.5435(5) 0.5070(18) 0.0741(15) 4.5(3)

Cu(3) Cu+1  24 0.4413(11) 0.4591(11) 0.424(2) 0.0400(7) 4.5(3)

O(10) O-2   6 0.4575(5) 0.5425(5) 0.25 0.308(4) 4.5(3)

C C   6 0.505(3) 0.495(3) 0.25 0.069(9) 3
```

CIF file the final structure of Cu_MAZ_CH4_290C

```
data_topas_cif_out

_chemical_name_mineral ?35keV?

_cell_length_a 18.1436(2)

_cell_length_b 18.1436(2)

_cell_length_c 7.59681(6)

_cell_angle_alpha 90

_cell_angle_beta 90

_cell_angle_gamma 120

_cell_volume 2165.75(5)

_symmetry_space_group_name_H-M P63/mmc

loop_

_symmetry_equiv_pos_as_xyz

 'x, y, z '

 '-x, -x+y, z+1/2 '

 '-x, -y, -z '

 '-x, -y, z+1/2 '

 '-x+y, -x, -z+1/2 '

 '-x+y, -x, z '

 '-x+y, y, -z+1/2 '
```

```
 '-x+y, y, z '
 '-y, -x, -z+1/2 '
 '-y, -x, z '
 '-y, x-y, -z+1/2 '
 '-y, x-y, z '
 'y, -x+y, -z '
 'y, -x+y, z+1/2 '
 'y, x, -z '
 'y, x, z+1/2 '
 'x-y, -y, -z '
 'x-y, -y, z+1/2 '
 'x-y, x, -z '
 'x-y, x, z+1/2 '
 'x, y, -z+1/2 '
 '-x, -x+y, -z '
 'x, x-y, -z+1/2 '
 'x, x-y, z '
loop_
_atom_site_label
_atom_site_type_symbol
```

```
_atom_site_symmetry_multiplicity

_atom_site_fract_x

_atom_site_fract_y

_atom_site_fract_z

_atom_site_occupancy

_atom_site_B_iso_or_equiv

Si(1) Si  24 0.73772(5) 0.64351(5) 0.54570(10) 1 2.400(13)

Si(2) Si  12 0.66775(9) 0.50713(8) 0.25 1 2.400(13)

O(1) O  12 0.72317(13) 0.72317(13) 0.5 1 3.25(3)

O(2) O  24 0.67601(12) 0.56204(9) 0.42585(18) 1 3.25(3)

O(3) O  12 0.83791(7) 1.67582(14) 0.5115(3) 1 3.25(3)

O(4) O  12 0.71028(15) 0.61435(15) 0.75 1 3.25(3)

O(5) O   6 0.73928(11) 0.4786(2) 0.25 1 3.25(3)

O(6) O   6 0.57352(15) 0.42648(15) 0.25 0.872(5) 3.25(3)

O(61) O   6 0.60594 0.39406 0.25 0.128(5) 3.25(3)

Cu(1) Cu+1   2 0.6666667 0.3333333 0.25 0.094(2) 5.0(3)

Cu(2) Cu+1  12 0.4578(4) 0.5422(4) 0.4938(15) 0.0791(14) 5.0(3)

Cu(3) Cu+1  24 0.569(2) 0.5451(19) 0.607(3) 0.0229(7) 5.0(3)

O(10) O-2   6 0.4549(6) 0.5451(6) 0.25 0.250(4) 5.0(3)

C C   6 0.5006(18) 0.4994(18) 0.25 0.121(10) 5.0(3)
```

Bibliography

[1] A. J. Knorpp, Direct Conversion of Methane to Methanol over Copper-Exchanged Zeolite Omega (MAZ), ETH Zürich, **2019**.

[2] A. J. Knorpp, A. B. Pinar, C. Baerlocher, L. B. Mccusker, N. Casati, M. A. Newton, S. Checchia, J. Meyet, D. Palagin, J. A. van Bokhoven, *Angewandte Chemie International Edition* **2020**, *60*, 5854–5858.

[3] J. Wieser, A. J. Knorpp, D. C. Stoian, P. Rzepka, M. A. Newton, J. A. van Bokhoven, *Angewandte Chemie International Edition* **2023**, DOI 10.1002/anie.202305140.

[4] M. A. Newton, S. Checchia, A. J. Knorpp, D. Stoian, W. Van Beek, H. Emerich, A. Longo, J. A. Van Bokhoven, *Catal Sci Technol* **2019**, *9*, 3081–3089.

[5] C. Bährle, V. Custodis, G. Jeschke, J. A. van Bokhoven, F. Vogel, *ChemSusChem* **2016**, *9*, 2397–2403.

[6] J. W. A. Fischer, F. Buttignol, A. Brenig, D. Klose, D. Ferri, V. Sushkevich, J. A. van Bokhoven, G. Jeschke, *Catal Today* **2024**, *429*, 114503.

[7] M. Mazur, *Anal Chim Acta* **2006**, *561*, 1–15.

[8] J. W. A. Fischer, A. Brenig, D. Klose, J. A. van Bokhoven, V. L. Sushkevich, G. Jeschke, *Angewandte Chemie International Edition* **2023**, DOI 10.1002/anie.202303574.

[9] A. Fitch, C. Dejoie, E. Covacci, G. Confalonieri, O. Grendal, L. Claustre, P. Guillou, J. Kieffer, W. De Nolf, S. Petitdemange, M. Ruat, Y. Watier, *J Synchrotron Radiat* **2023**, *30*, 1003–1012.

[10] T. Donath, D. Šišak Jung, M. Burian, V. Radicci, P. Zambon, A. N. Fitch, C. Dejoie, B. Zhang, M. Ruat, M. Hanfland, C. M. Kewish, G. A. Van Riessen, D. Naumenko, H. Amenitsch, G. Bourenkov, G. Bricogne, A. Chari, C. Schulze-Briese, *J Synchrotron Radiat* **2023**, *30*, 723–738.

[11] S. J. A. Figueroa, C. Prestopino, *J Phys Conf Ser* **2016**, *712*, DOI 10.1088/1742-6596/712/1/012012.

[12] P. Rzepka, C. V. Colin, T. C. Hansen, A. J. Knorpp, V. Nassif, M. A. Newton, I. Puente Orench, J. A. van Bokhoven, D. Wardecki, **2021**, DOI 10.5291/ILL-DATA.5-22-788.

[13] R. Czoch, *Appl Magn Reson* **1996**, *10*, 293–317.

[14] J.-S. Yu, J. Y. Kim, C. W. Lee, S. J. Kim, S. B. Hongc, L. Kevand, **1997**, 4211–4219.

[15] E. A. Velasco-Rozo, L. M. Ballesteros-Rueda, V. G. Baldovino-Medrano, *J Am Soc Mass Spectrom* **2021**, *32*, 2135–2143.

[16] M. A. Newton, A. J. Knorpp, V. L. Sushkevich, D. Palagin, J. A. van Bokhoven, *Chem. Soc. Rev* **2020**, *49*, 1449–1486.

[17] K. T. Dinh, M. M. Sullivan, K. Narsimhan, P. Serna, R. J. Meyer, M. Dincă, Y. Román-Leshkov, *J Am Chem Soc* **2019**, *141*, 11641–11650.
